# Co‐Translational Deposition of *N* ^6^‐Acetyl‐*L*‐Lysine in Nascent Proteins Contributes to the Acetylome in Mammalian Cells

**DOI:** 10.1002/advs.202403309

**Published:** 2024-12-04

**Authors:** Dingyuan Guo, Nan Li, Xiaoyan Zhang, Runxin Zhou, Jie He, Xiao‐Ping Ding, Weixing Yu, Fuqiang Tong, Sibi Yin, Yu Wang, Xin Xu, Long Wang, Mingzhu Fan, Shan Feng, Ke Liu, Ke Tang, Zhuqing Ouyang, Yusong R Guo, Yugang Wang

**Affiliations:** ^1^ Department of Biochemistry and Molecular Biology School of Basic Medicine Tongji Medical College and State Key Laboratory for Diagnosis and Treatment of Severe Zoonotic Infectious Diseases Huazhong University of Science and Technology Wuhan Hubei 430030 China; ^2^ Department of Pathogen Biology, School of Basic Medicine, Tongji Medical College Huazhong University of Science and Technology Wuhan Hubei 430030 China; ^3^ Hubei Institute for Drug Control Wuhan Hubei 430075 China; ^4^ School of Chemistry and Chemical Engineering and Hubei Key Laboratory of Bioinorganic Chemistry and Materia Medica Huazhong University of Science and Technology Wuhan Hubei 430074 China; ^5^ Mass Spectrometry & Metabolomics Core Facility The Biomedical Research Core Facility Center for Research Equipment and Facilities Westlake University Hangzhou Zhejiang 310024 China; ^6^ Key Laboratory of Structural Biology of Zhejiang Province School of Life Sciences Westlake University Hangzhou Zhejiang 310024 China; ^7^ Department of Biostatistics School of Public Health Cheeloo College of Medicine Shandong University Jinan 250000 China; ^8^ Cell Architecture Research Center Huazhong University of Science and Technology Wuhan Hubei 430030 China

**Keywords:** co‐translational modification, KARS, protein acetylation

## Abstract

*N* ^*6*^‐acetyl‐*L*‐*lysine* residue is abundant in dietary protein but little is known about its potential influences on the diet‐consumers. Herein, it is reported that Lysyl‐tRNA synthetase (KARS) mediates co‐translational deposition of diet‐derived *N* ^6^‐acetyl‐*L*‐lysine (AcK) in nascent proteins to contribute to the acetylome in cells. Acetylated dietary protein is a direct source of AcK that can widely and substantially regulate the acetylome in multiple organs of mice. By analyzing the mechanisms underlying AcK contributing to the acetylome in mammalian cells, it is found that KARS can utilize AcK as an alternative substrate to produce *N* ^6^‐acetyl‐ *l* ‐lysyl‐tRNA. The crystal structure of KARS in complex with AcK at 2.26 Å resolution shows that AcK shares the same substrate‐binding pocket as *L*‐lysine, allowed by a sidechain flip of Tyr499. The generated *N* ^6^‐acetyl‐*L*‐lysyl‐tRNA introduces AcK into growing nascent polypeptide and results in protein acetylation, including the regions buried inside folded proteins that are post‐translational modification (PTM)‐inaccessible and functionally important. This undocumented protein modification mechanism is inherently different from PTM and termed as co‐translational modification (coTM). It is expected to extend the repertoire of acetylome and improve the understanding of protein modification mechanisms in cells.

## Graphical abstract

This study reports that Lysyl‐tRNA synthetase (KARS) can mediate the co‐translational deposition of *N* ^6^‐acetyl‐*L*‐lysine (AcK) in nascent proteins to contribute to the acetylome in cells. This undocumented protein modification mechanism is inherently different from PTM (post‐translational modification) and termed as co‐translational modification (coTM). CoTM can widely and substantially regulate the acetylome in multiple organs of mammals, resulting in an extended repertoire of protein modification.

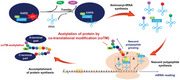

## 1 Introduction

*N* ^6^‐acetyl‐ *l* ‐lysine residue is abundant in dietary proteins. In husbandry, lysine acetylation naturally occurs during the growth of animals and crops.^[^ ^1^, ^2^, ^3^ ^]^ In the food industry, lysine acetylation is intensively added to dietary proteins via chemical reactions, aiming to improve the physicochemical and functional properties of commercialized proteins,^[^ ^4^, ^5^, ^6^ ^]^ such as coffee whiteners,^[^ ^4^ ^]^ flavoring agents for roasted meat,^[^ ^4^ ^]^ fish myofibrillar protein,^[^ ^7^ ^]^ and oat protein isolate.^[^ ^8^ ^]^ In mammals, such as humans, lysine acetylation is a type of frequent and important protein post‐translational modification (PTM) regulating protein functions and cell physiologies.^[^ ^9^ ^]^ For example, lysine acetylation of core histones is a class of central mechanisms regulating chromatin function, such as gene expression and DNA damage replication.^[^ ^9^ ^]^ It responds to extracellular and intracellular signals and precisely regulates cellular processes to maintain homeostasis.^[^ ^9^ ^]^ Dysregulation of histone acetylation is tightly associated with human diseases, such as cancer.^[^ ^9^ ^]^ Therefore, it is worth asking whether and how intensively consuming acetylated dietary proteins would influence the acetylome of diet‐consumers. *N* ^6^‐acetyl‐ *l* ‐lysine (AcK) has been identified as a metabolite with little‐known function in mammals.^[^ ^10^, ^11^, ^12^ ^]^ It is tempting to speculate that AcK could be released from dietary proteins, be absorbed by dietary protein consumers, and influence the acetylome of dietary protein consumers via uncovered mechanisms.

To address the questions above, we fed mice with deuterium‐labeled acetylated protein and confirmed that acetylated dietary protein is a direct source of AcK that can widely and substantially contribute to the acetylome in organs of mice, including the liver, brain, and lung. AcK in a culture medium can be rapidly transported into cells. Lysyl‐tRNA synthetase (KARS) can alternatively utilize AcK as the substrate to generate *N* ^6^‐acetyl‐ *l* ‐lysyl‐tRNA, which subsequently participates in protein synthesis and introduces AcK into growing nascent polypeptide, resulting in protein acetylation during the process of translation. As such, we termed this undocumented mechanism for protein modification as co‐translational modification (coTM). The coTM‐generated protein acetylation is chemically identical to the one deposited by PTM‐mechanism. However, because the coTM‐acetylation occurs prior to the folding of polypeptides, it can result in acetylation in folded and buried regions that are PTM‐inaccessible, affecting protein functions via reprofiling inter‐residue interactions inside proteins.

## 2 *N* ^6^‐acetyl‐ *l* ‐Lysine Residues in Dietary Protein Contribute to the Acetylome of Diet‐Consumer

**Figure 1:**
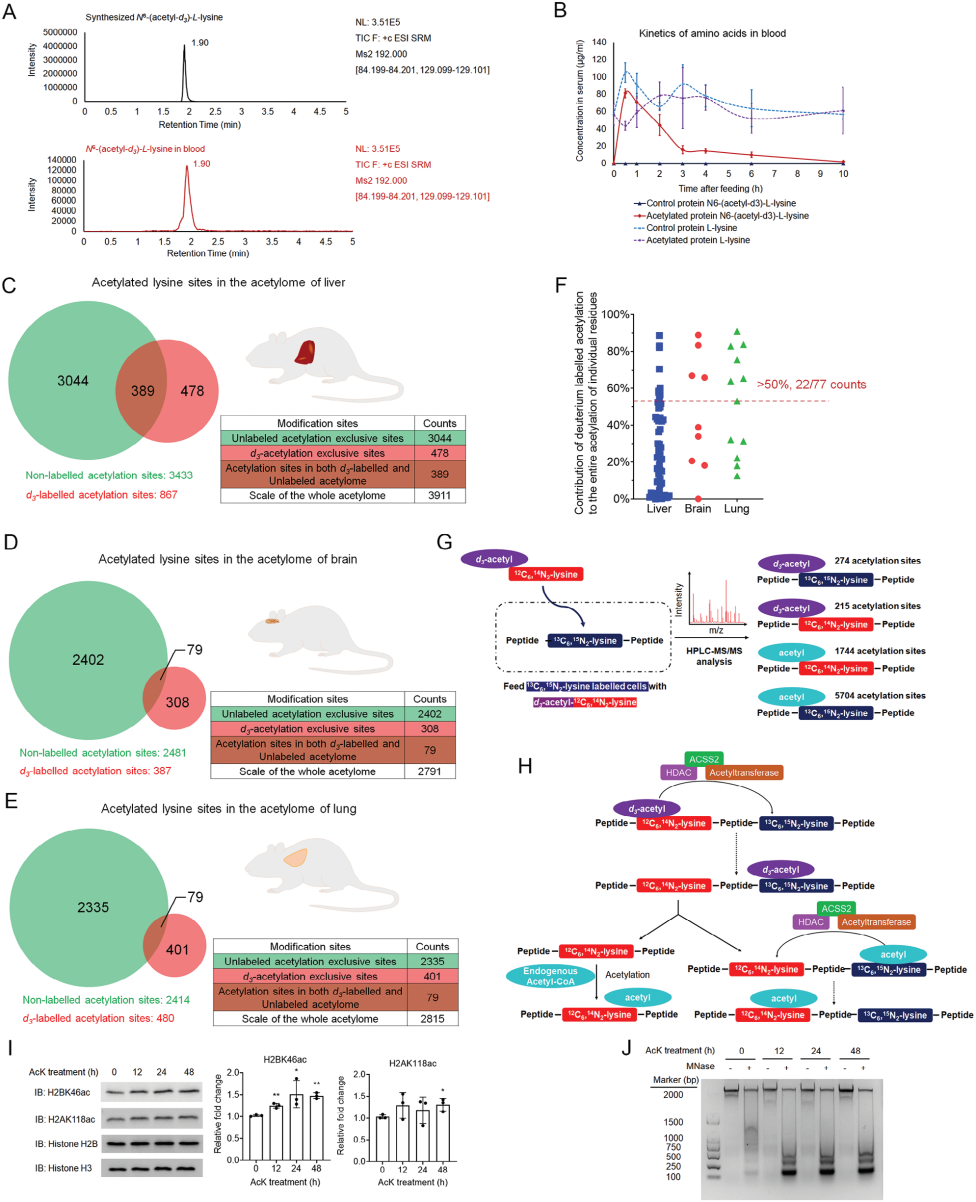
*N* ^6^‐acetyl‐*l*‐lysine residues in dietary proteins contribute to the acetylome of diet‐consumer. A) Identification of *d_3_*‐AcK in the blood of mice fed with *N* ^6^‐(acetyl‐*d_3_*)‐ *l* ‐lysine‐protein. HPLC‐MS/MS profiles of chemically synthesized *d_3_*‐AcK (black) and *d_3_*‐AcK in blood samples (red). The *y*‐axis indicates the signal intensity, the *x*‐axis indicates the retention time (min). B) The kinetics of *d_3_*‐AcK and lysine in the blood of mice fed with *N* ^6^‐(acetyl‐*d_3_*)‐l‐lysine‐protein and control protein, respectively. Each data point is presented as the means ± s.d. of five mice (*n =* 5). C–E) Acetylome of organs in mice fed with *N* ^6^‐(acetyl‐*d_3_*)‐l‐lysine‐protein. The number of deuterium‐labeled and unlabeled acetylation sites in the C) liver, D) brain, and E) lung was identified by HPLC‐MS/MS. F) Contribution of deuterium‐labeled acetylation to individual acetylated lysine residues. To avoid the biases inherent in the use of antibody‐based enrichment methods that employed prior to the proteomic analysis,^[^ ^39^ ^]^ only the pairs of deuterium‐labeled acetylation peptides and unlabeled acetylation peptides with the same features (length, amino acid sequence, modifications, and modification sites) were used for the quantitative analyses. Details for calculating the contribution of deuterium‐labeled acetylation to the entire acetylation of each lysine residue are described in the section on materials and methods. G) Analysis of the acetylome in ^13^C_6_,^15^N_2_‐ *l* ‐lysine‐labeled cells treated by *d_3_*‐AcK. The left panel shows the proposed scheme of *d_3_*‐AcK incorporating in the ^13^C_6_,^15^N_2_‐l‐lysine‐labeled proteome. The right panel shows the scheme of the identified peptides in the analyses. The number of identified acetylation positions is presented. Detailed information about the identified acetylation positions is listed in Table S4 (Supporting Information). H) The scheme of local transferring of acetyl‐moiety results in different combinations between acetyl‐groups and lysine residues. I) AcK treatment upregulates acetylation of core histones. The cultured HeLa cells were treated with 1 mM AcK for a series of time points. The acetylation level of histone H2A K118 and histone H2B K46 in the treated cells were analyzed by immunoblotting assays with indicated antibodies. Representative images of triplicate experiments are shown. The statistical analyses of these immunoblotting results are provided on the right side. Two‐sided *t*‐test analyses were conducted. The data are presented as the means ± s.d. of three independent experiments (*n =* 3). *p < 0.05, **p < 0.01. J) AcK treatment induces decondensation of chromatin in cells. Nuclei were isolated from cultured cells treated with 5 mM AcK for a series of time points. The nuclei were then incubated with MNase, by which the genomic DNA in the decondensated chromatin regions was digested. The genomic DNA with and without MNase digestion was separated in 1.5% DNA electrophoresis agarose gel and visualized by DNA dye staining. Representative images of triplicate experiments are shown.

To understand whether the *N* ^6^‐acetyl‐ *l* ‐lysine residues in dietary proteins could influence the acetylome of diet‐consumer, we produced and fed deuterium‐labeled acetylated protein (*N* ^6^‐(acetyl‐*d*_*3*_)‐ *l* ‐lysine‐protein) to mice (Figure S1A,B, Supporting Information). Blood samples were collected and analyzed by performing high‐performance liquid chromatography‐tandem mass spectrometry (HPLC‐MS/MS). Identification of *N* ^6^‐(acetyl‐d_3_)‐ *l* ‐lysine (d_3_‐AcK) in these blood samples demonstrated that acetylated dietary protein is a direct source of AcK in protein‐consumer (**Figure** **1**A,B; Figure S1C, Supporting Information). We next collected liver, brain, and lung from the studied mice to analyze acetylation profiles. Besides unlabeled acetylation sites, we identified 867, 387, and 480 deuterium‐labeled acetylation sites in the liver, brain, and lung tissues, respectively (Figure 1C–E; Table S1, Supporting Information). We then analyzed the relative abundance of deuterium‐labeled acetylation to the entire acetylation of certain lysine residues that were identified in both deuterium‐labeled acetylome and unlabeled acetylome. In the studied acetylation profiles, about 28.6% of acetylated lysine residues were majorly affected by deuterium‐labeled acetylation (accounts for >50% of certain acetylation sites) (Figure 1F). The maximum contribution of deuterium‐labeled acetylation to individual acetylated lysine residues reached 90.9% (Figure 1F; Table S2, Supporting Information), indicating that diet‐derived AcK might substantially contribute to the acetylation of certain lysine residues in the acetylome of protein‐consumer. By performing biological processes enrichment analysis, we found that deuterium‐labeled acetylation occurs on 556 proteins that are enriched in 32 biological processes in the studied liver tissue (Figure S2 and Table S3, Supporting Information), 314 proteins that are enriched in 15 biological processes in the studied brain tissue (Figure S3 and Table S3, Supporting Information), and 333 proteins that are enriched in 9 biological processes in the studied lung tissue (Figure S4 and Table S3, Supporting Information), respectively. These findings suggest that food‐derived AcK can widely influence the acetylome and biological processes in mammals. Notably, most of the deuterium‐labeled acetylation‐enriched biological processes and unlabeled acetylation‐enriched processes are overlapped (Figures S2–S4, Supporting Information), suggesting that diet‐contributed acetylome is regulated to match physiological states.^[^ ^13^ ^]^ These findings, together, ascertain that acetylated dietary protein can release AcK and contribute to the acetylomes in diet consumers.

To understand how AcK contributes to the acetylome in mammalian cells, we labeled the proteome of cultured cells with ^13^C_6_,^15^N_2_‐l‐lysine and treated the labeled cells with deuterium‐labeled AcK (*N* ^6^‐(acetyl‐d_3_)‐ *l* ‐^12^C_6_,^14^N_2_‐lysine, d_3_‐AcK) (Figure S5A,B, Supporting Information). Proteomic analyses identified 274 *N* ^6^‐(acetyl‐d_3_)‐^13^C_6_,^15^N_2_‐lysine‐labeled acetylation sites, 1744 *N* ^6^‐acetyl‐^12^C_6_,^14^N_2_‐lysine acetylation sites, and 215 *N* ^6^‐(acetyl‐d_3_)‐^12^C_6_,^14^N_2_‐lysine‐labeled acetylation sites (Figure 1G; Table S4, Supporting Information). Two explanations were proposed for these findings. First, d_3_‐AcK can release and separately contribute deuterium‐labeled acetyl‐moiety and L‐lysine,^[^ ^14^ ^]^ resulting in random combinations of deuterium‐labeled acetyl‐moiety and L‐lysine in the proteome. However, feeding d_3_‐AcK to the ^13^C_6_,^15^N_2_‐l‐lysine‐labeled cells did not significantly contribute to intracellular L‐^12^C_6_,^14^N_2_‐lysine (Figure S6A, Supporting Information). No deuterium labeled acetyl‐CoA was identified either (Figure S6B, Supporting Information). We therefore tempted to speculate the second possible mechanism that d_3_‐AcK is directly incorporated in the ^13^C_6_,^15^N_2_‐l‐lysine‐labeled proteome. Acetyl‐CoA synthetase 2 (ACSS2) can directly facilitate the local transfer of acetyl groups between lysine residues of proteins.^[^ ^15^ ^]^ So the local transfer of deuterium‐labeled and unlabeled acetyl‐moiety might result in combinations between d_3_‐acetyl‐moiety, acetyl‐moiety, ^13^C_6_,^15^N_2_‐l‐lysine, and ^12^C_6_,^14^N_2_‐l‐lysine (Figure 1H).

There were 215 *N* ^6^‐(acetyl‐d_3_)‐^12^C_6_,^14^N_2_‐l‐lysine residues identified in the labeling studies (Figure 1G), including the Lys‐34 and Lys‐46/47 of histone H2B, Lys‐118/119 and Lys‐124 of histone H2A. Consistently, AcK treatment induced acetylation of these lysine residues and chromatin decondensation (Figure 1I,J), which was in line with the report that histone hyperacetylation induces chromatin decondensation.^[^ ^16^ ^]^ AcK treatment showed a minor influence on the level of pan‐acetylation in cells (Figure S6C, Supporting Information). However, the antagonization of deacetylases unleashed AcK‐induced upregulation of pan‐acetylation in cells (Figure S6D, Supporting Information), suggesting that deacetylases might serve as surveillance mechanisms harnessing AcK‐induced acetylation in cells.

## 3 *N* ^6^‐acetyl‐*L*‐lysine is an alternative substrate of KARS

Neumann et al. established an Escherichia coli strain expressing engineered pyrrolysyl‐tRNA synthetase (PylRS) that accommodates AcK in the catalytic pocket,^[^ ^17^ ^]^ allowing AcK to be directly deposited into nascent polypeptides to produce proteins with acetylation. If the deposition of d_3_‐AcK in proteomics naturally occurs in mammalian cells, it is worth asking whether the wild‐type of KARS could directly utilize AcK as an alternative substrate to produce *N* ^6^‐acetyl‐ *l* ‐lysyl‐tRNA.

**Figure 2:**
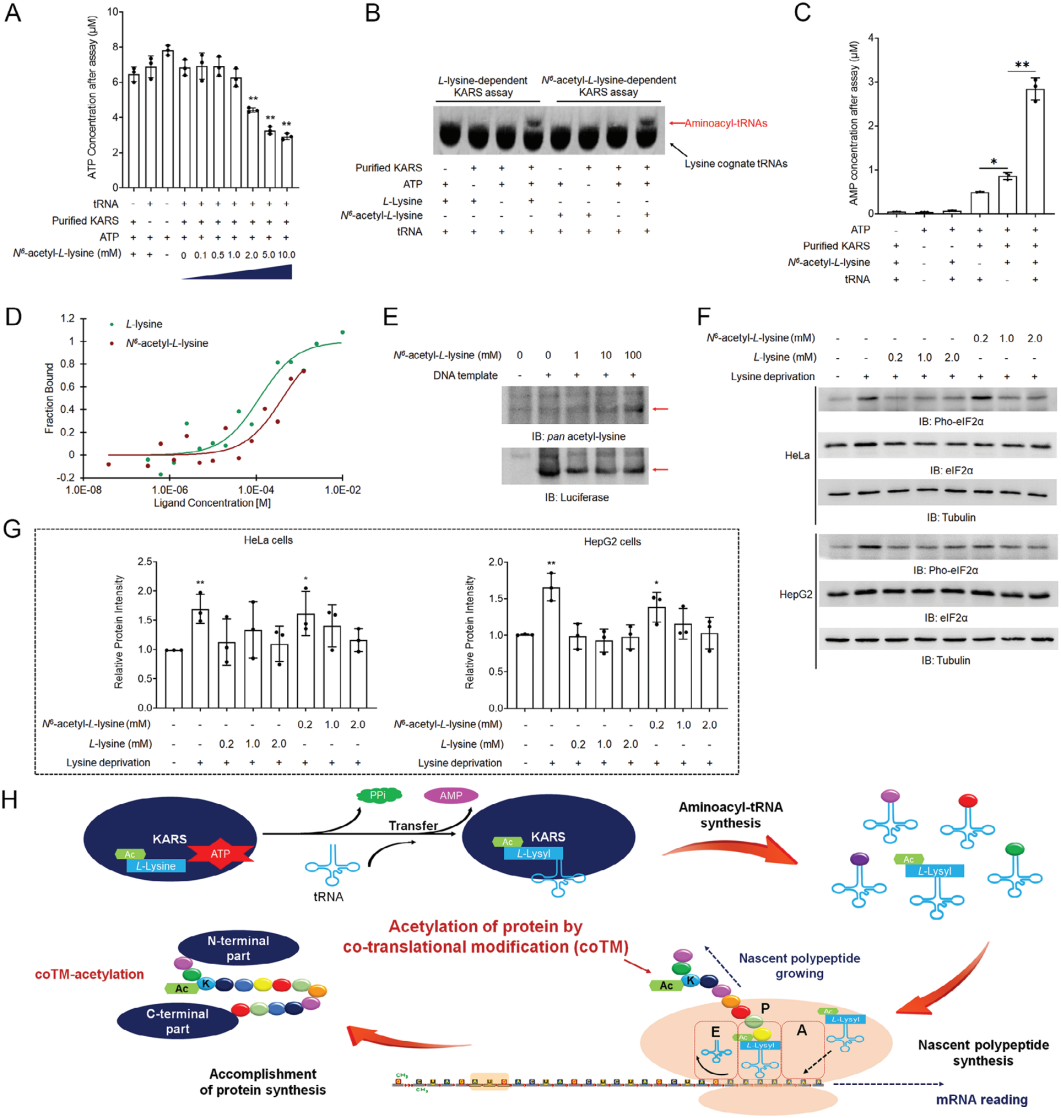
*N* ^6^‐acetyl‐l‐lysine is an alternative substrate of KARS. A) In vitro activity assay of KARS utilizing AcK as substrate. Purified KARS was incubated with ATP, AcK, and tRNAs. The ATP consumption in the assays were quantitatively analyzed. Two‐sided *t*‐test analyses were conducted. The data are presented as the means ± s.d. of three independent experiments (*n =* 3). **p < 0.01. B) KARS‐catalyzed generation of aminoacyl‐tRNAs. Northern blotting analysis of lysine cognate tRNAs by using fluorescence‐labeled single‐strand DNA probe that specifically recognizes lysine cognate tRNAs. The free lysine cognate tRNA and aminoacyl‐tRNAs are indicated with black and red arrows, respectively. Representative images of triplicate experiments are shown. C) In vitro activity assays of KARS utilizing AcK as substrate. Purified KARS was incubated with ATP, AcK, and tRNAs. The AMP production in the assays were quantitatively analyzed. Two‐sided *t*‐test analyses were conducted. The data are presented as the means ± s.d. of three independent experiments (*n =* 3). *p < 0.05, **p < 0.01. D) MicroScale Thermophoresis (MST) assays measuring the Kd of KARS binding to *L*‐lysine and AcK. The dose‐response curves for KARS binding to *L*‐lysine (green) or AcK (dark red) are presented. The *y*‐axis indicates the fraction of bound target as a function of ligand concentration, the *x*‐axis indicates the concentration of substrate in each titration. E) The addition of AcK in a cell‐free protein expression system results in the acetylation of synthesized protein. The synthesized protein and its acetylation level were analyzed by performing immunoblotting assays with the indicated antibodies. Representative images of triplicate experiments are shown. F,G) AcK diminishes the suppressive effect of lysine deprivation on protein synthesis in cells. Immunoblotting assays were performed with indicated antibodies (F). Representative images of triplicate experiments are shown. The statistical analyses of these immunoblotting results are provided (G). Two‐sided *t*‐test analyses were conducted. The data are presented as the means ± s.d. of three independent experiments (*n =* 3). *p < 0.05, **p < 0.01. H) Scheme of the KARS‐mediated co‐translational deposition of AcK in nascent protein resulting in protein acetylation.

To produce L‐lysyl‐tRNA, KARS first utilizes L‐lysine and ATP as substrates to produce L‐lysyl‐AMP, then transfers the L‐lysyl‐group to tRNA, generating L‐lysyl‐tRNA and releasing AMP (Figure S7A, Supporting Information). By incubating purified KARS with tRNAs, ATP, and AcK, we observed AcK‐dependent ATP consumption in the in vitro assays (**Figure** **2**A), suggesting that KARS can catalyze the production of *N* ^6^‐acetyl‐ *l* ‐lysyl‐AMP. Northern blotting assays following KARS‐mediated in vitro reactions revealed a mobility shift of lysine cognate tRNAs in both the AcK and L‐lysine dependent assays (Figure 2B; Figure S7B, Supporting Information). The shifted lysine cognate tRNAs in the L‐lysine dependent assays is L‐lysyl‐tRNA (Figure 2B). Compared to the mechanism of the KARS‐K reaction (Figure S7A, Supporting Information), the shifted lysine cognate tRNAs in AcK‐dependent assay (Figure 2B), together with the production of AMP in the AcK‐dependent assay (Figure 2C), strongly suggest that KARS can transfer the *N* ^6^‐acetyl‐ *l* ‐lysyl‐moiety from *N* ^6^‐acetyl‐ *l* ‐lysyl‐AMP to tRNA to generate *N* ^6^‐acetyl‐ *l* ‐lysyl‐tRNA.

To further understand AcK‐dependent KARS catalysis, we did the steady‐state kinetics analyses and found that the maximum velocity (Vmax) and Michaelis Constant (K_m_) of AcK‐dependent KARS reaction is 0.1564 (95% confidence interval: 0.113 to 0.243) µM·min^−1^ and 2.234 (95% confidence interval: 0.783 to 6.759) µM (Table S5, Supporting Information), respectively. Previous studies reported that KARS can catalyze the formation of L‐lysyl‐AMP, which can attack the *N* ^6^‐amino of *L*‐lysine to generate L‐lysyl‐ *l* ‐lysine and release AMP.^[^ ^18^, ^19^ ^]^ For this reason, we observed substantial ATP consumption and AMP production by KARS in the absence of tRNAs (Figure S7C,D, Supporting Information). Despite that, no study reported a similar reaction on AcK, tRNA‐independent AMP production was also observed in KARS‐AcK reaction (Figure 2C). As such, the Km and Vmax of the KARS‐K and KARS‐AcK assays cannot be precisely compared by these assays. MicroScale Thermophoresis (MST) assays revealed that the disassociation constant (Kd) of KARS binding to AcK (407 ± 291 µM) is higher than that of L‐lysine (118 ± 55.5 µM) (Figure 2D).

KARS‐catalyzed L‐lysyl‐tRNA is a vehicle introducing L‐lysine into growing nascent polypeptides in protein synthesis. *N* ^6^‐acetyl‐ *l* ‐lysyl‐tRNA is likely to have a similar role in introducing AcK into growing polypeptides and generating nascent proteins bearing acetylated‐lysine residues. This hypothesis was first supported by the finding that the addition of AcK in a mammalian cell‐free protein expression system can dose‐dependently upregulate the acetylation of synthesized protein (Figure 2E). Addition of d_3_‐AcK led to the identification of the synthesized protein in the cell‐free protein expression system (Figure S7E, Supporting Information). No AcK‐derived lysine was identified within 2 h in living cells (Figure S6A, Supporting Information). No AcK‐derived acetyl‐CoA could be generated within 2 h either (Figure S6B, Supporting Information). We, therefore, consider no acetyl‐moiety could be released from AcK and post‐translationally contribute to protein acetylation in the mammalian cell‐derived protein synthesis system. The elevated acetylation of the synthesized protein by AcK treatment is highly likely to result from the direct incorporation of AcK in the newly synthesized protein (Figure 2E). Secondly, lysine deprivation can suppress protein synthesis and induce phosphorylation of eIF2α.^[^ ^20^, ^21^ ^]^ Addition of AcK dose‐dependently reduced the phosphorylation of eIF2α that was induced by lysine deprivation (Figure 2F,G), strongly suggesting that AcK can be a substitute for L‐lysine to initiate protein synthesis that was suppressed by lysine deprivation. No upregulated phosphorylation of eIF2α was observed in cells cultured with 0.2 mM L‐lysine (Figure 2F,G). By comparison, a higher concentration of AcK was required to reduce lysine deprivation‐induced phosphorylation of eIF2α (Figure 2F,G). These findings suggest that KARS is more efficient in utilizing L‐lysine than AcK.

These above results collectively demonstrate that KARS can utilize AcK to generate *N* ^6^‐acetyl‐ *l* ‐lysyl‐tRNA, which can introduce AcK into growing polypeptides in protein synthesis and generate nascent proteins with acetylation (Figure 2H).

## 4 The Molecular Basis of KARS Utilizing *N* ^6^‐acetyl‐ *l* ‐Lysine as Substrate

**Figure 3:**
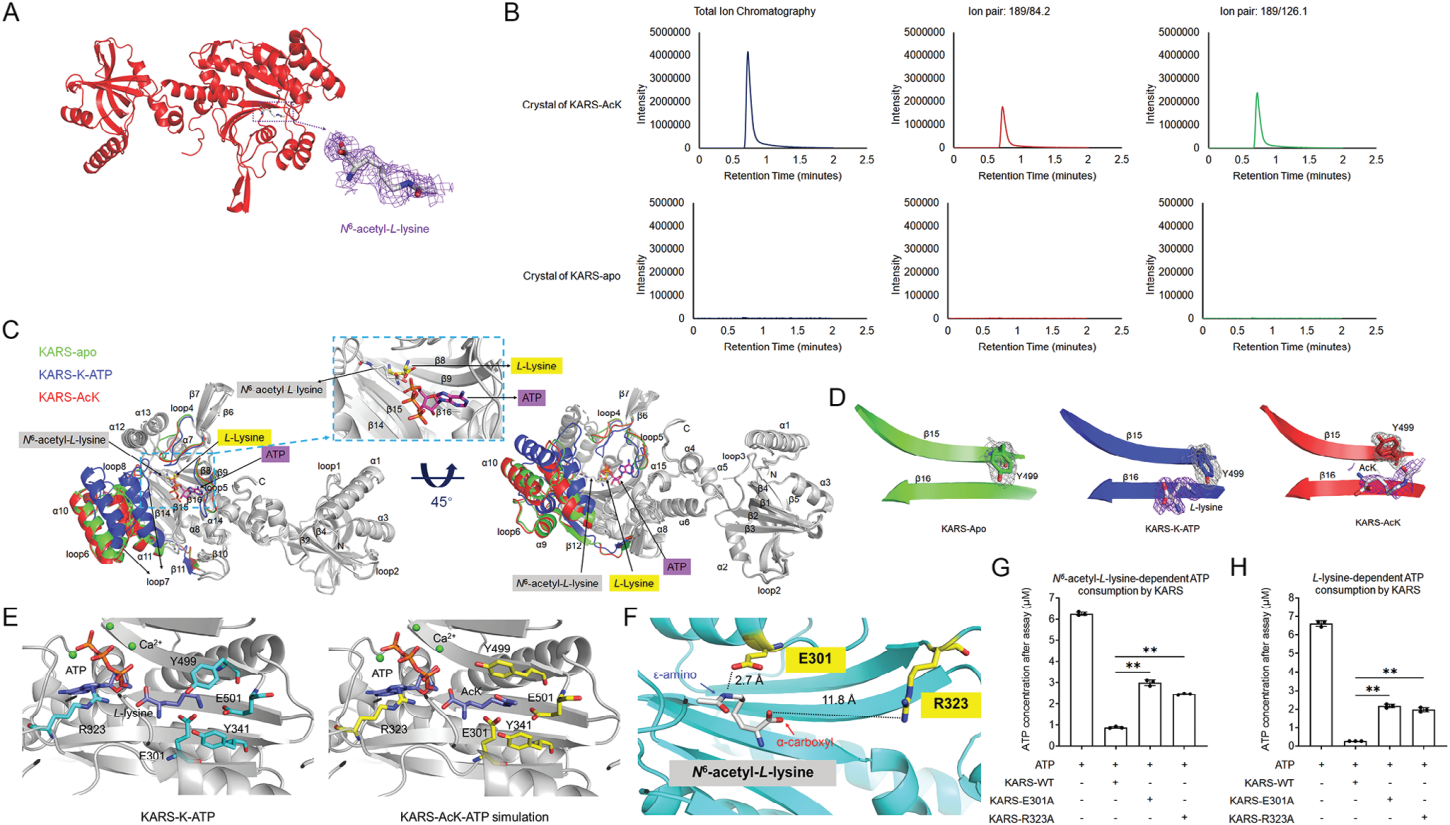
The molecular basis of KARS utilizing *N* ^6^‐acetyl‐*L*‐lysine as substrate. A). Co‐crystal structure of KARS in complex with AcK. Cartoon drawing of KARS‐AcK structure with a zoomed‐in view of AcK. The ligand is shown as sticks, along with their 2*Fo*‐*Fc* electron density map (purple mesh, contoured at 0.9*σ*). B). Identification of AcK in the crystals. Small compound from the crystals of KARS‐apo and KARS‐AcK was extracted and identified by HPLC‐MS/MS with indicated ion pairs. The *y*‐axis indicates the signal intensity, the *x*‐axis indicates the retention time (min). C) Superimposition analysis of the structures of KARS‐apo, KARS‐K‐ATP, and KARS‐AcK. Only the regions showing structural differences between the KARS‐apo (green), KARS‐K‐ATP (blue), and KARS‐AcK (red) are colored and labeled. *L*‐lysine (yellow) and ATP (violet) in the structure of KARS‐K‐ATP and AcK (gray) in the structure of KARS‐AcK are shown as sticks. The substrate‐binding pocket is zoomed in and labeled. D) Comparison of Tyr499 conformation in KARS structures with different ligands. The three structures shown from left to right are: apo structure of human KARS (green, this study), human KARS‐K‐ATP complex (blue, PDB ID 3BJU), and human KARS‐AcK complex (red, this study). The β sheet 15 and β sheet 16 are shown in the cartoon. The electron density map for the Tyr499 residues (colored sticks) are shown as a dark gray mesh (1.0 σ contour level). The electron density map for the *L*‐Lysine and AcK (gray sticks) are shown as a purple‐blue mesh (1.0 σ contour level). E) Comparison of the substrate binding pockets in KARS‐K‐ATP complex (left, PDB ID: 3BJU) and KARS‐ATP‐AcK complex simulation structure (right). The KARS is shown as a gray cartoon. The five clamps (Glu301, Arg323, Tyr341, Tyr499, and Glu501) are displayed as cyan sticks in KARS‐ATP‐K complex, and yellow sticks in the simulation structure. The ATP, *L*‐lysine, and AcK are shown as slate sticks, and calcium ions are shown as green spheres. F) The position of AcK in the structure of KARS‐AcK. The AcK surrounding regions of KARS‐AcK structure are shown as cartoons in cyan. AcK and KARS‐E301‐R323 residues are shown as sticks in gray and yellow, respectively. The distance between the ε‐amino moiety of AcK and the carboxyl moiety of KARS‐E301 is measured and labeled. The distance between the α‐carboxyl moiety of AcK and KARS‐R323 is measured and labeled. G,H), KARS mutants abolished the catalytic activities. Purified KARS wide‐type and mutants were incubated with ATP, tRNAs, and G) AcK or H) *L*‐lysine. The ATP consumption in the assays were measured and quantitatively analyzed. Two‐sided *t*‐test analyses were conducted. The data are presented as the means ± s.d. of three independent experiments (*n =* 3). **p < 0.01.

The molecular basis of KARS utilizing L‐lysine to generate L‐lysyl‐tRNA has been revealed by the crystal structure of KARS in a complex with lysine and ATP (PDB ID: 3BJU).^[^ ^22^ ^]^ The binding pocket of L‐lysine is highly compact, with the ε‐amino group clamped by a pair of negatively‐charged residues Glu‐301 and Glu‐501 and a pair of aromatic residues Tyr‐341 and Tyr‐499, to ensure the strict substrate specificity of KARS, leaving no space for an extra acetyl group (Figure S8A, Supporting Information). However, the enzymatic activity assays and MST analysis strongly suggest that AcK is a substrate of KARS, illustrating an unearthed mechanism underlying KARS‐AcK interaction. To validate this speculation, we incubated KARS, AcK, and ATP for structural analysis using X‐ray crystallography. We obtained two crystal forms for the apo and complex forms of KARS, respectively. The structures were determined by molecular replacement with the known structure of the KARS‐lysine‐ATP complex (KARS‐K‐ATP, PDB ID: 3BJU). To avoid model bias, the lysine and ATP molecules were removed from the model for molecular replacement. We then refined the final crystal structures to 2.55 Å resolution for the apoprotein (PDB ID: 8HYR) and 2.26 Å resolution for the complex (PDB ID: 8XP4) (Table S6, Supporting Information). In the substrate‐binding pocket of the complex structure, we found electron density that fits AcK (**Figure** **3**A). We next extracted the small compound from the crystals of the complex and confirmed it as AcK by performing HPLC‐MS/MS analysis (Figure 3B). These results collectively demonstrate that KARS can accommodate AcK in its substrate‐binding pocket. The complex structure was thus termed as KARS‐AcK.

The overall structures of KARS‐apo and KARS‐AcK are similar to the KARS‐K‐ATP complex (PDB ID: 3BJU) (Figure 3C), with root‐mean‐square deviations of 0.772 and 0.791 Å, respectively. Both AcK and L‐lysine share similar positions in the substrate‐binding pocket of KARS (Figure 3C). By binding to L‐lysine and ATP, loop 7 and loop 8, together with their adjacent helices α9, α10, and α11 (residues N395‐T455) were drawn closer to the binding pocket (Figure S8B, Supporting Information), resulting in a compacted catalytic pocket to facilitate the interaction between substrates for catalytic reaction (Figure S8B, Supporting Information).^[^ ^23^ ^]^ KARS‐AcK accommodates AcK in the substrate‐binding pocket. However, likely because of the absence of ATP, its catalytic pocket remains in a “standby state” which could allow the access of ATP for catalytic reaction. Therefore, the conformation surrounding the substrate‐binding pocket of KARS‐AcK is similar to that of KARS‐apo (Figure S8C, Supporting Information),

Compared to the KARS‐apo and KARS‐K‐ATP structures, the side chain of Tyr‐499 in the KARS‐AcK complex is flipped away from the center of the substrate binding pocket (Figure 3D; Figure S8D, Supporting Information). This rotameric change creates additional space for the acetyl group of AcK while keeping other parts of the substrate binding pocket intact (Figure S8A,D, Supporting Information). ATP binding is essential for KARS‐catalyzed reaction. To understand whether ATP and AcK binding can induce KARS to form similar compact conformation, the KARS‐K‐ATP structure (PDB ID: 3BJU) was utilized to perform the following simulation analysis. First, the side‐chain of Tyr‐499 was flipped as the one we observed in KARS‐AcK structure (PDB ID: 8XP4) (Figure 3E). Second, the L‐lysine in the KARS‐K‐ATP structure was replaced by AcK, whose α‐carboxyl‐moiety and ε‐amino group aligns with that of L‐lysine in the KARS‐K‐ATP structure (PDB ID: 3BJU) (Figure 3E). The result strongly suggests that ATP accommodates well in the simulated KARS‐AcK‐ATP structure. The L‐lysine interacting residues, such as Arg‐323, Tyr‐499, Glu‐301, Tyr‐341, and Glu‐501, in the substrate‐binding pocket of KARS, also interact with the L‐lysine portion of AcK (Figure 3E).

Glu‐301 and Arg‐323 are critical residues for the catalytic activity of KARS (Figure S8E, Supporting Information).^[^ ^22^, ^24^ ^]^ In the complex of KARS‐K‐ATP, an electrostatic interaction between the ε‐amino group of L‐lysine and the carboxyl group of Glu‐301 is critical for KARS recognizing L‐lysine (Figure S8E, Supporting Information).^[^ ^22^, ^24^ ^]^ In terms of AcK, the positive charge of its ε‐amino group is neutralized by acetylation, so that the critical electrostatic interaction is disrupted. However, in the structure of KARS‐AcK, a hydrogen bond between the ε‐amino group of AcK and the carboxyl group of Glu‐301 can be formed as a substitution of the electrostatic interaction (Figure 3F). Replacement of the Glu‐301 residue with alanine (KARS‐E301A) disrupts the interaction of Glu‐301 with both L‐lysine and AcK. As a result, this mutation significantly reduced the activities of KARS in both AcK and L‐lysine‐dependent assays (Figure 3G,H). Arg‐323 interacts with both ATP and the α‐carboxyl‐moiety of L‐lysine in the structure of KARS‐K‐ATP (Figure S8E, Supporting Information). It is critical for KARS to catalyze the formation of L‐lysyl‐AMP.^[^ ^22^ ^]^ In the structure of KARS‐AcK, the Arg‐323 residue is 11.8 Å from the α‐carboxyl‐moiety of AcK (Figure 3F), suggesting no interaction between Arg‐323 and AcK in the complex of KARS‐AcK. However, the replacement of Arg‐323 residue with alanine (KARS‐R323A) significantly suppressed the activities of KARS in both AcK and L‐lysine‐dependent assays (Figure 3G,H). These results, together with the interaction between Arg‐323 and ATP in the structure of KARS‐K‐ATP (Figure S8E, Supporting Information), suggest that the Arg‐323 residue might also interact with ATP in KARS‐AcK reaction. The structure of KARS‐K‐ATP contains both ATP and L‐lysine so that its catalytic domain is compact enough to perform catalysis (Figure S8B, Supporting Information). Structure simulation analysis revealed that both ATP and AcK accommodate well in the compacted catalytic pocket of KARS (Figure 3E),^[^ ^23^ ^]^ illustrating that substrate‐induced conformational change is likely to occur in KARS‐AcK reaction (Figure S8F, Supporting Information).

These structural analyses, together with the findings from activity assays, collectively demonstrate that KARS can directly utilize AcK to produce *N* ^6^‐acetyl‐ *l* ‐lysyl‐tRNA via a molecular basis similar to the one in L‐lysyl‐tRNA generation. *N* ^6^‐acetyl‐ *l* ‐lysyl‐tRNA can co‐translationally introduce AcK in polypeptide synthesis and produce acetylated nascent proteins in mammalian cells.

## 5 Deposition of *N* ^6^‐acetyl‐*l*‐Lysine in the Buried Regions Of Proteins

**Figure 4:**
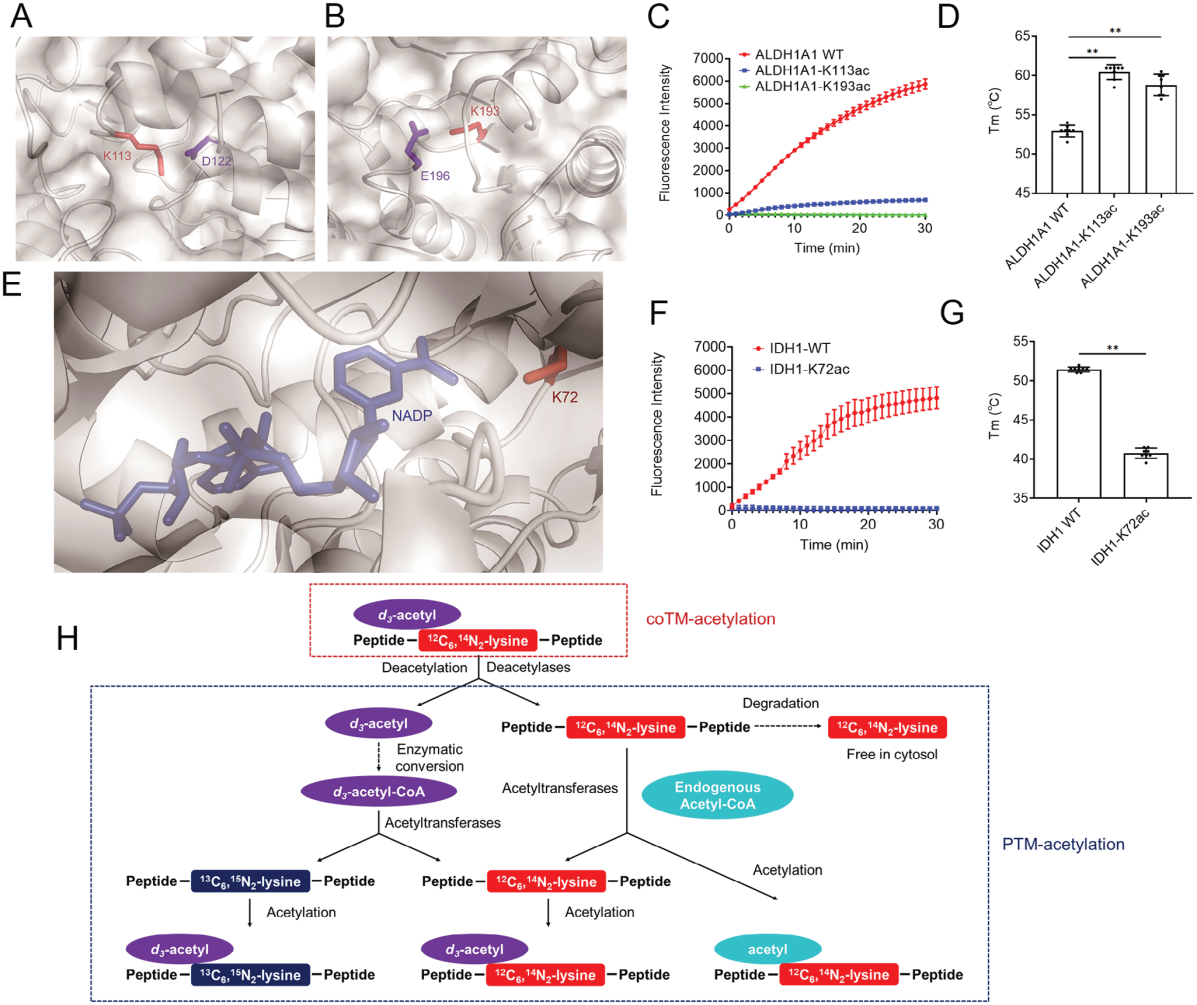
Deposition of *N* ^6^‐acetyl‐l‐lysine in the buried regions of proteins. A,B) Zoomed‐in and transparent view of mouse ALDH1A1 structure (PDB ID: 7YOB). The Lys‐113 residue (A) and Lys‐193 residue (B) that localize in the buried regions of ALDH1A1 are illustrated. The acetylation‐affected lysine residues are shown as sticks and highlighted in red. The negatively charged Asp‐122 and Glu‐196 that interact with Lys‐113 and Lys‐193 are shown as sticks and highlighted in blue, respectively. C) Acetylation of Lys‐113 and Lys‐193 reduces the activity of ALDH1A1. The enzymatic activity assays of ALDH1A1 wild‐type, ALDH1A1‐K113ac, and ALDH1A1‐K193ac were studied by measuring the NADH production in each assay. Each data point is presented as the means ± s.d. of three independent assays (*n =* 3). D) Acetylation of Lys‐113 and Lys‐193 changes the thermostability of ALDH1A1. The temperatures of melting of ALDH1A1 wild‐type, ALDH1A1‐K113ac, and ALDH1A1‐K193ac were measured by performing thermofluor shift assays. Two‐sided *t*‐test analyses were conducted. Each data point is presented as the means ± s.d. of eight independent assays (*n =* 8). **p < 0.01. E) Zoomed‐in and transparent view of mouse IDH1 structure (PDB ID: 5YZH). The Lys‐72 residue that localizes in the buried regions of IDH1 is shown as sticks and highlighted in red. The substrate NADP is highlighted in blue. F) Acetylation of Lys‐72 reduces the activity of IDH1. The enzymatic activity assays of IDH1 wild‐type and IDH1‐K72ac were studied by measuring the NADPH production in each assay. Each data is presented as the means ± s.d. of three independent assays (*n =* 3). G) Acetylation of Lys‐72 reduces the thermostability of IDH1. The temperatures of melting of IDH1 wild‐type and IDH1‐K72ac were measured by performing thermofluor shift assays. Two‐sided *t*‐test analyses were conducted. Each data point is presented as the means ± s.d. of eight independent assays (*n =* 8). **p < 0.01. H) Scheme of the possible pathways for the cross‐talk between coTM‐acetylation and PTM‐acetylation.

In PTM‐acetylation, acetyltransferases physically contact the lysine residues to catalyze acetylation of substrate lysine residues, so that only the lysine residues that are on the surface and structurally allow the access of acetyltransferases are possible to be modified.^[^ ^25^ ^]^ In the studied acetylomes, we found 63 deuterium‐labeled acetylation sites localized in the buried regions of proteins that are inaccessible to PTM mechanisms (Table S7, Supporting Information). For instance, we identified two coTM‐acetylated lysine residues that are buried inside the protein of retinal dehydrogenase (ALDH1A1, UniProt ID: P24549, Lys‐113, and Lys‐193) (Figure S9A,B, Supporting Information). Acetylation can neutralize the positive charge of Lys‐113 and Lys‐193 which might disrupt their interactions with Asp122 and Glu196, respectively (**Figure** **4**A,B). To unambiguously determine the effect of these buried Lys‐113 acetylation and Lys‐193 acetylation on ALDH1A1, we utilized genetic code expansion orthogonal systems to incorporate acetyl‐lysine into recombinant ALDH1A1 to create Lys113‐acetylated ALDH1A1 (ALDH1A1‐K113ac) and Lys193‐acetylated ALDH1A1 (ALDH1A1‐K193ac) (Figure S9C, Supporting Information). In vitro enzymatic activity assays revealed that acetylation of Lys‐113 or Lys‐193 abrogates ALDH1A1 activity (Figure 4C). Addition of AcK to cultured HEK293 cells also reduced ALDH1A1 activity (Figure S9D, Supporting Information). By performing thermofluor shift assays, we further found that both ALDH1A1‐K113ac and ALDH1A1‐K193ac have a significantly higher melting temperature (*T*_m_) than that of ALDH1A1 without acetylation (Figure 4D), strongly suggesting that acetylation of these buried lysine residues might reprofile inter‐residue interactions and change the thermostability of ALDH1A1.

Cytoplasmic isocitrate dehydrogenase (IDH1, UniProt ID: O88844) is another instance. CoTM‐acetylated lysine residue (Lys‐72) localizes inside the folded structure and around the substrate (NADP) binding pocket (Figure 4E; Figure S9E, Supporting Information). We expressed and purified recombinant proteins of IDH1 wide‐type and IDH1‐K72ac (Figure S9F, Supporting Information). The recombinant IDH1‐K72ac protein is activity deficient (Figure 4F). Consistently, AcK supplement reduced the activity of IDH1 protein in cultured mammalian cells (Figure S9G, Supporting Information). Moreover, Thermofluor shift assays revealed that IDH1‐K72ac has a lower Tm than that of IDH1 protein without Lys‐72 acetylation (Figure 4G). These findings strongly suggest that coTM‐acetylation of Lys‐72 residue is deleterious for IDH1 protein activity and thermostability.

The above instances illustrate that coTM‐acetylation of buried lysine residues might reprofile inter‐residues interactions of proteins. It could theoretically result in a large number of protein misfolding and induce unfolded protein response (UPR) to maintain proteostasis.^[^ ^26^ ^]^ Phosphorylation of eIF2α and cleavage of ATF6 are indicators of UPR activation. However, AcK treatment only slightly upregulated phosphorylation of eIF2α (Figure S9H,I, Supporting Information). No cleaved ATF6 was observed in the same set of samples (Figure S9H,I, Supporting Information). These findings suggest that AcK treatment might not be able to generate a sufficient amount of dysfunctional proteins to significantly activate the UPR pathway in mammalian cells. While more studies are needed, we expect future discovery of mechanisms surveilling “unwanted” acetylation in the process of protein synthesis.

Taken together, the present study demonstrates that AcK can be co‐translationally incorporated in nascent proteins and contribute to the acetylome in mammalian cells. KARS can alternatively utilize AcK to produce *N* ^6^‐acetyl‐ *l* ‐lysyl‐tRNA that co‐translationally deposits AcK into nascent polypeptide synthesis. Compared with the well‐known PTM mechanism that occurs after protein synthesis,^[^ ^27^ ^]^ this undocumented mechanism leads to protein acetylation during the process of protein translation, it is therefore termed as co‐translational modification (coTM). AcK is a metabolite that can be directly derived from acetylated dietary protein. It can result in coTM‐acetylation naturally and widely in multiple organs. The present study provides us with a new understanding of an undescribed mechanism regulating protein modification and extends the repertoire of acetylome in cells.

## 6 Discussion

Protein acetylation is an important and frequent type of PTM that regulates multiple biological processes in mammals.^[^ ^13^ ^]^ Here, we show that, in addition to PTM, co‐translational incorporation of AcK in nascent protein is an alternative pathway contributing to protein acetylation in mammals. This mechanism deposits acetylation in proteins prior to the folding of nascent polypeptide, so that buried and PTM mechanism‐inaccessible regions of proteins can also be modified by this newly discovered mechanism. The coTM process occurs naturally and widely in mammals, we, therefore, consider it as an important supplementary pathway to diversify protein modification profiles.

In the ^13^C_6_,^15^N_2_‐l‐lysine‐labeled cells treated by d_3_‐AcK, acetylation sites with different isotope‐labeling profiles were identified (Figure 1G). These findings strongly suggest that the profiles of PTM‐acetylation and coTM‐acetylation might cross‐talk with each other via the following possible mechanisms. First, direct deposition of AcK in nascent proteins results in the identification of *N* ^6^‐(acetyl‐d_3_)‐ *l* ‐^12^C_6_,^14^N_2_‐lysine in the ^13^C_6_,^15^N_2_‐l‐lysine‐labeled cells (Figures 1G and 4H). Second, the deuterium‐labeled acetyl‐moiety can be removed by deacetylase, leaving the L‐^12^C_6_,^14^N_2_‐lysine residues that could be re‐acetylated by PTM mechanisms.^[^ ^14^, ^28^ ^]^ The *N* ^6^‐acetyl‐^12^C_6_,^14^N_2_‐lysine sites were therefore identified (Figures 1G and 4H). Third, the removed acetyl‐moiety could theoretically be recycled as d_3_‐acetyl‐CoA. It can be utilized in the acetylation of other lysine residues,^[^ ^29^, ^30^ ^]^ so that *N* ^6^‐(acetyl‐d_3_)‐^13^C_6_,^15^N_2_‐lysine sites were identified (Figures 1G and 4H). Lastly, degradation of both *N* ^6^‐(acetyl‐d_3_)‐ *l* ‐^12^C_6_,^14^N_2_‐lysine and *N* ^6^‐acetyl‐^12^C_6_,^14^N_2_‐lysine bearing proteins can release nonlabeled‐lysine, so that delayed release of lysine in the d_3_‐AcK treated ^13^C_6_,^15^N_2_‐l‐lysine‐labeled cells might be identified (Figure 4H).

Acetyl‐CoA is the major vehicle transferring acetyl‐moiety between lysine residues in proteome.^[^ ^13^ ^]^ But no deuterium‐labeled acetyl‐CoA was identified in the present study (Figure S6B, Supporting Information), so the local transfer of deuterium‐labeled and unlabeled acetyl‐moiety was proposed as the major reason for the combinations between d_3_‐acetyl‐moiety, acetyl‐moiety, ^13^C_6_,^15^N_2_‐l‐lysine and ^12^C_6_,^14^N_2_‐l‐lysine (Figure 1H). Indeed, intracellular acetyl‐CoA pools are affected by the dynamic activities of acetyltransferase, deacetylases, and various metabolic enzymes that contribute to acetyl‐CoA production and consumption.^[^ ^31^, ^32^, ^33^ ^]^ As such, there were at least three explanations for the failure in identifying deuterium labeled acetyl‐CoA in this study. First, the local transfer of acetyl‐moiety between lysine residues limits the release of acetate and acetyl‐CoA as free molecules in cells. For example, Acetyl‐CoA synthetase 2 (ACSS2) has been proposed to recapture acetate released by HDACs and to provide a local pool of acetyl‐CoA for protein acetylation.^[^ ^34^ ^]^ It can directly facilitate the local transfer of acetyl groups between lysine residues of proteins,^[^ ^15^ ^]^ so that a limited amount of acetate and acetyl‐CoA can be released from the repository, despite that recent estimates suggest that substantial amount of acetate could be produced from deacetylation of proteins for generation of acetyl‐CoA.^[^ ^35^ ^]^ Second, the recycling rate of the acetyl groups derived from the d_3_‐AcK incorporated peptides are limited, so only a small amount of deuterium‐labeled acetyl‐CoA can be generated. Third, although the acetyl‐group could be recycled from the d_3_‐AcK incorporated peptides, the generated acetyl‐CoA might be rapidly utilized by enzymes for metabolism or acetyltransferases for protein acetylation, so that no deuterium‐labeled acetyl‐CoA was able to be quantitatively analyzed by HPLC‐MS/MS.

This study demonstrates that diet‐derived AcK can directly contribute to the acetylome in the mammalian system. While acetylome is determinant in maintaining physiological homeostasis in cells and organs,^[^ ^9^ ^]^ it is premature, at this stage, to claim any physiological impact of daily consumption of acetylated dietary protein on health. In the mammalian system, the protein acetylation profile is monitored and maintained by both acetyltransferases and deacetylases.^[^ ^36^ ^]^ Deposition of AcK in nascent protein, theoretically, occurs randomly. However, unfavorable acetylation would likely be removed by deacetylases to ensure that the acetylomes match the physiological homeostasis of cells.^[^ ^25^, ^37^ ^]^ This assumption is supported by the observation that most of the deuterium‐labeled acetylation‐enriched biological processes overlap with unlabeled acetylation‐enriched processes (Figures S2–S4, Supporting Information). As such, limited influence on the pan‐acetylation in cells was observed (Figure S6C, Supporting Information). Dysfunction of the deacetylases‐dependent surveillance mechanism unleashed AcK‐contributed acetylation in cells (Figure S6D, Supporting Information). Deacetylases inhibitors are important therapeutics in clinics,^[^ ^38^ ^]^ so the consumption of acetylated dietary protein might need to be alerted by patients taking deacetylases inhibitors for disease treatment. Besides, the potential effects of long‐term and intensive consumption of acetylated dietary protein on health remain elusive.

## 7 Experimental Section

### Materials

Primary antibodies for immunoblotting: β‐actin (mouse, Cat#AC004, lot#3500100006, Abclonal, Wuhan, China), Acetylated‐lysine (rabbit, Cat#9441S, lot#15, Cell Signaling Technology, MA, USA), α‐Tubulin (mouse, Cat# AC012, lot#9100012001, Abclonal, Wuhan, China), p‐eIF2alpha(S51)(rabbit, Cat#D9G8, lot#8, Cell Signaling Technology, MA, USA), eIF2alpha(rabbit, Cat#D7D3, lot#9, Cell Signaling Technology, MA, USA), ATF6(mouse, Cat#66563‐1, lot#10031826, Proteintech, Wuhan, China). Luciferase (rabbit, Cat#A18259, lot#3501634001, Abclonal, Wuhan, China), Flag‐tag (mouse, Cat#AE005, lot#9200005002, Abclonal, Wuhan, China). The SILAC DMEM medium (Cat#88425) were purchased from Thermo Fisher Scientific (Shanghai, China) and prepared according to the manufacturer's guidelines. A fluorescent probe for detecting lysine cognate tRNA probe (5′Cy5.5‐ATT AAG AGT CAT ACG CG) was synthesized and purchased from Sangon Biotech (Shanghai, China). Nucleic acid gel stain Solargreen (Cat#G5570) was purchased from Solarbio (Beijing, China). Northern blot hybridization solution (Cat#AM8663) and nylon membrane (Cat#AM10100) were purchased from Thermo Fisher Scientific (Shanghai, China). Diethyl Pyrocarbonate (Cat#V900882) and tRNA from brewer′s yeast (Cat#10109525001) were purchased from Sigma‐Aldrich (Shanghai, China). *N* ^6^‐acetyl‐ *l* ‐lysine (Cat#N159376) were purchased from Aladdin (Shanghai, China). Six‐week‐old female C57BL/J mice were purchased from Hubei Bainte Biological Technology Co., Ltd.

### Ethics Statement

All animal experiments were performed in accordance with the animal protocols and regulations approved by the HUST Experimental Animal Ethics Committee of Huazhong University of Science and Technology (Approval Number: 2019‐S2145).

### Cell Culture and Stable Isotope Labeling

HeLa cells were kindly provided by Dr. Weidong Xie at Tsinghua University. The cells were analyzed by STR sequencing for cell line authorization. The cells were cultured in Dulbecco's modified Eagle's medium (Cat#SH30285.01, Cytiva Life Sciences, MA, USA) supplemented with 10% fetal bovine serum (Cat#086‐150, WISENTINC, Quebec, Canada) and 1% penicillin and streptomycin (Cat#BL505A, biosharp, Beijing, China). The cultured cells were kept at 37 °C in the cell culture incubator (CLM‐170B‐8‐NF, ESCO, Singapore) with 5% CO_2_.

To label the proteome of cultured cells, cells were labeled and maintained in SILAC DMEM medium that was supplemented with 1mM ^13^C_6_^15^N_2_‐l‐lysine (Cat#608041, Sigma‐Aldrich, Shanghai, China), 10% dialyzed fetal bovine serum, and 1% penicillin and streptomycin. After ten cell doublings, the labeling efficiency was >99.5%, assessed by the mass spectrometer.

### Synthesis of *N* ^6^‐(acetyl‐d_3_)‐ *l* ‐lysine

To obtain *N* ^6^‐(acetyl‐d_3_)‐ *l* ‐lysine, L‐Lysine was first dissolved in NaHCO_3_ aqueous solution. To the given solution, CuSO_4_·5H_2_O in water and NaHCO_3_ were added. The mixture was cooled to 0 °C by an ice bath and acetic anhydride‐d_6_ (Cat#175641, Sigma‐Aldrich, Shanghai, China) was added dropwise. After stirring at room temperature for 12 h, a pale blue suspension was formed. After filtration, the pale blue solid was suspended in water, then 8‐hydroxyquinoline was added. The suspension was stirred for 10 h, and the color changed from blue to yellow. After filtration, the filtrate was washed with ethyl acetate. The aqueous phase was freeze‐dried to a solid product (yield: 28%). The products were identified by the ^1^H NMR spectrum.

### Acetylation of Soy Proteins

The acetylation of soy proteins was performed according to the protocol that was previously reported.^[^ ^40^ ^]^ Briefly, soy protein (Cat#9010‐10‐0, Shanghai Macklin Biochemical Co., Ltd, Shanghai, China) was homogenized with 0.1M carbonate buffer, pH 8.3, for 3 h at room temperature. Acetic anhydride‐d_6_ (Cat#175641, Sigma‐Aldrich, St. Louis, USA) was gradually added to the soy protein isolate solution (anhydride/protei*n =* 0.6–1 g/g) and incubated for 1 h. The solution pH was adjusted with 2.5 M NaOH to be in the range of 8.0–8.5. After the pH was stabilized, the solution was kept at room temperature for 2 h and then dialyzed with double‐distilled water at 4 °C for 48h to remove excess anhydride. The dialyzed protein was freeze‐dried and stored at −20 °C.

### Acetyl‐peptide Enrichment and Identification

The ^13^C_6_^15^N_2_‐l‐lysine‐labeled HeLa cells (>6 × 10^8^) were treated with 10mM *N* ^6^‐(acetyl‐d_3_)‐ *l* ‐lysine for 48h. Then the cells were collected and washed three times with ice‐cold PBS. Mice were daily fed with 0.1g *N* ^6^‐(acetyl‐d_3_)‐ *l* ‐lysine‐protein for 5 days. Organs and tissues were collected and washed three times with ice‐cold PBS and stored at −80 °C for later analysis.

Collected cells or tissue samples (>0.2g each) were lysed and homogenized with urea lysis buffer (20 mM HEPES pH 8.0, 9 M urea, 1 × protease inhibitor cocktail (MedChemExpress (MCE), NJ, USA. Cat#: HY‐K0010), 1 × phosphatase inhibitor cocktail (MCE, NJ, USA. Cat#: HY‐K0021), and 1 × deacetylase Inhibitor Cocktail (MCE, NJ, USA. Cat#: HY‐K0030)). Lysates were sonicated (60‐watt output with 6 bursts of 10 s each, ice‐bath for 30 s between each burst) to shear genomic DNA. The protein concentrations were determined using the BCA protein assay kit. 20 mg protein from each sample was processed for further procedures.

Acetylated peptides were enriched following the PTMScan kit (Cell Signaling Technologies, Danvers, MA, USA) protocol with some modifications. Briefly, proteins were reduced with 5 mM dithiothreital at room temperature for 60 min followed by alkylation with 20 mM iodoacetamide in the dark for 30 min at room temperature. Proteins were diluted 4‐fold with 20 mM HEPES pH 8.0 and then digested with trypsin at the enzyme‐to‐substrate ratio of 1:75 overnight at room temperature with gentle mixing. Peptides from the digestion reactions were desalted using an Oasis HLB cartridge and lyophilized. To enrich acetyl‐peptides, the sample was incubated with PTMScan acetyl‐lysine motif immunoaffinity beads overnight at 4 °C. The beads were washed twice with immunoaffinity purification buffer and three times with mass spectrometry‐grade water. Then the enriched acetyl‐peptides were eluted with 100 ul of 0.15% TFA and lyophilized.

For identification of acetyl‐peptides by LC‐MS/MS. The enriched acetyl‐peptides were analyzed with the Thermo EASY‐nLC1200 integrated nano‐HPLC system that was directly interfaced with the Thermo Q Exactive HF‐X mass spectrometer. Peptides in the chromatographic system were eluted with solvent A (0.1% formic acid) and solvent B (80% acetonitrile and 0.1% formic acid) at a flow rate of 0.300 µL min^−1^. Each sample was analyzed nine times (6.5µL of the sample per run). The mass spectrometer was operated in the data‐dependent acquisition mode using the Xcalibur 4.1 software. A single full‐scan mass spectrum in the Orbitrap (400–1800 m/z, 60 000 resolution) was followed by 20 data‐dependent MS/MS scans at 30% normalized collision energy. Each mass spectrum was analyzed using the Thermo Xcalibur Qual Browser and Proteome Discoverer for the database search.

Quantitative analysis of coTM‐acetylation was only performed by using the pairs of coTM‐acetylated peptides and PTM‐acetylation peptides with the same features (length, amino acid sequence, modifications, and modification sites). The contribution of coTM‐acetylation to the entire acetylation of each lysine residue was calculated by using the equation, $\;x\;=\frac{a}{a+b},$ where x presents the contribution of coTM‐acetylation to the entire acetylation of each lysine residue, a presents the abundance of the coTM‐acetylated peptide calculated in the MS system, b presents the abundance of the PTM‐acetylated peptide that had the same profile with that of coTM‐acetylated peptide.

### Quantitative Analysis of *N* ^6^‐(acetyl‐d_3_)‐ *l* ‐lysine in Serum

Blood samples were collected from mice fed with 0.05 g *N* ^6^‐(acetyl‐d_3_)‐ *l* ‐lysine‐protein or control protein (without acetylation treatment). The blood samples were collected from the mouse's retro‐orbital sinus at the indicated time points. The serum was isolated by centrifuging the blood at 3000 rpm for 15 min. 50 µL serum was obtained at each time‐point from each mouse. The metabolites were extracted twice with 80% methanol in water. Two extracts from the same sample were combined and dried by a Vacufuge Concentrator System (Eppendorf 5301, Eppendorf, Germany).

The amino acids in mice serum were quantitatively analyzed using LC‐MS/MS. 50 µL of methanol/water (1:4, v/v) was applied for reconstitution. The final reconstitution samples (injection volume: 2 µL) were injected into a 100 × 4.6 mm 3.5 µm high‐performance liquid chromatography column (XBridge Amide, Waters, USA). The mobile phase A consisted of 5 mM ammonium acetate in 0.1% formic acid solution, and mobile phase B was methanol. Data acquisition was performed using an Ultimate 3000 HPLC system interfaced with a TSQ Quantum Access MAX triple quadrupole Mass Spectrometry (Thermo Fisher Scientific, Waltham, MA, USA).

### Quantitative Analysis of *N* ^6^‐(acetyl‐d_3_)‐ *l* ‐lysine in HeLa Cell

To analyze the intracellular concentration of *N* ^6^‐(acetyl‐d_3_)‐ *l* ‐lysine, the cultured cells were treated with 1 mM *N* ^6^‐(acetyl‐d_3_)‐ *l* ‐lysine and collected at the indicated time points. The collected cells were washed three times with ice‐cold PBS. The metabolites were extracted with 80% methanol in water and collected following 15 000 centrifugation for 20 min. The metabolites were included in the supernatant that was then dried by Vacufuge Concentrator System (Eppendorf 5301, Eppendorf, Germany). The final reconstitution samples were injected into a 100 × 4.6 mm 3.5 µm high‐performance liquid chromatography column (XBridge Amide, Waters, USA). The mobile phase consisted of ammonium acetate and formic acid in both aqueous and organic phases. Data acquisition was performed using a UHPLC–LTQ/Orbitrap MS (Thermo Fisher Scientific, Waltham, MA, USA) via an Ion Trap Orbitrap positive ion mode.

### Plasmids, Protein Expression and Purification

The KARS_70–580_ gene was cloned into pET‐20b vector with C‐terminal 6 × His tag and overexpressed in the Escherichia coli BL‐21 strain. Protein expression was induced with 0.2 mM isopropyl β‐D‐1‐thiogalactopyranoside (IPTG) at 16 °C for 20 h. Then the bacteria were harvested and resuspended in a lysis buffer (20 mM Tris‐HCl pH 8.0, 350 mM NaCl, 20 mM imidazole, 1 mM PMSF, 1 mM benzamidine, and 10 mM β‐Mercaptoethanol). The cells were lysed by “JNBIO” Low‐Temperature Ultra‐High‐Pressure Continuous Flow Cell Disrupter (JN‐Mini Pro, JNBIO, Guangzhou, China), and the soluble supernatant was collected by centrifugation at 25000 g for 1 h at 4 °C. The supernatant was passed through a gravity column with 2 mL Ni Focurose 6FF IMAC (HZ1003‐5, HUIYAN Bio, Wuhan, China) that had previously been equilibrated with lysis buffer. Protein was then purified using a Resource Q 6 mL column (17‐1179‐01, GE Healthcare, Uppsala, Sweden) and a Superdex 200 Increase 10/300 column (28‐9909‐44, GE Healthcare, Uppsala, Sweden) equilibrated with 25 mM HEPES pH 7.5, 150 mM NaCl and 5 mM DTT. The peak fractions containing the protein were pooled and concentrated in centrifugal concentrators (CLVG01230, Merck Millipore, Cork, Ireland) to a final concentration of 30 mg mL^−1^ for crystallization. Plasmids expressing KARS‐R323A and KARS‐E301 were made by site‐directed mutagenesis.

The mouse IDH1 and ALDH1A1 genes were cloned into a pColdI expression vector with an N‐terminal hexahistidine tag separately and overexpressed in Escherichia coli T7 Express cell line (BC206‐01, Biomed, Beijing, China). Protein expression was induced with 0.2 mM isopropyl β‐D‐1‐thiogalactopyranoside at 16 °C for 20 h. Then the bacteria were harvested and the protein was purified with Ni Focurose 6FF IMAC (HZ1003‐5, HUIYAN Bio, Wuhan, China) and eluted with elution buffer 2 (20 mM Tris‐HCl, pH 7.5, 500 mM NaCl, 300 mM imidazole, pH8.0, 10% glycerol, 5 mM β‐Mercaptoethanol). Then the protein was concentrated with centrifugal concentrators (UFC901096, Merck Millipore, Cork, Ireland), and loaded onto a Superose 6 Increase 10/300 GL gel‐filtration column (29‐0915‐96, GE Healthcare, Uppsala, Sweden) equilibrated with 20 mM HEPES, pH 7.5, 200 mM NaCl, 2 mM DTT, 10% glycerol. The purity of the fractions containing the protein was checked by SDS‐PAGE analysis.

### Expression and Purification of Recombination Proteins of IDH1‐K72ac, ALDH1A1‐K113ac, and ALDH1A1‐K193ac

Plasmids of pEVOL‐AcKRS (Addgene: 137976) and pCold carrying mouse IDH1 or ALDH1A1 gene with an amber stop codon at the designed position for acetylation were transformed into BL21(DE3) E. coli strain. A Single colony was picked and inoculated into 2YT medium supplemented with 100 µg mL^−1^ ampicillin and 50 µg mL^−1^ chloramphenicol at 37 °C overnight. A 20 mL volume of the overnight cultured bacteria was transferred in 500 mL 2YT medium. When OD600 reached 0.6–0.8, 20 mM nicotinamide (NAM) and 100 mM acetyl‐lysine were added and incubated at 16 °C for 30 min. Then 0.5 mM IPTG and 2 mM L‐arabinose were added to induce IDH1 or ALDH1A1 expression at 16 °C for 16–20 h. Cells were collected after induction, and purified by using a Ni‐affinity chromatography column.

### Microscale Thermophoresis

The microscale thermophoresis (MST) assay was performed according to the protocol that was previously described.^[^ ^41^ ^]^ Briefly, the assay was performed on a NanoTemper Monolith NT.115 with blue/red filters (NanoTemper Technologies GmbH, Munich, Germany). Purified recombinant KARS protein was labeled by His‐Tag Labeling Kit RED‐tris‐NTA 2nd generation (MO‐L018, NanoTemper Technologies GmbH, Germany) according to the manufacturer's instructions. For MST binding affinity assays, lysine or acetyl‐lysine was used for a 16‐step serial dilution in MST assay buffer (30 mM HEPES, pH 7.4, 150 mM NaCl, 30 mM KCl, 40 mM MgCl_2_, 1 mM DTT and 0.05% Tween‐20) with 10 µl volume in each sample. Labeled KARS (10 µL) was added to a series of gradient concentrations of L‐lysine (10 µL, from 20 mM to 0.61 µM, 16 concentrations, dilution factor: 2) and acetyl‐lysine solution (10 µL, from 2.5 mM to 0.0763 µM, 16 concentrations, dilution factor: 2). The mixture (20 µL) was then transferred into a Monolith standard capillary and loaded onto the NanoTemper Monolith to measure the fluorescence from each trace. Data analyses were performed using the NanoTemper analysis software.

### Recombinant Protein Activity Assays

KARS activity assays were performed according to the protocol that was previously described.^[^ ^42^ ^]^ Briefly, the reaction mixture containing 30 mM Tris‐Cl pH 7.5, 150 mM NaCl, 30 mM KCl, 40 mM MgCl_2_, 1 mM DTT, 10 µM ATP, 3 mg mL^−1^ tRNA, and 10 mM lysine or *N* ^6^‐acetyl‐*L*‐lysine were started by adding 20 µg KARS (WT/E301A/R323A). Enzymatic activities of KARS wild‐type and mutants were determined by measuring AMP production and ATP consumption with AMP‐Glo Assay Kit (Cat#V5011, Promega, Madison, USA) and ATP Bioluminescent Assay Kit (Cat#FLAA, Sigma‐Aldrich, Shanghai, China) or ATP Assay Kit (Cat#S0026, Beyotime, Shanghai, China), respectively.

IDH1 activity assays were performed according to the protocol that was previously reported.^[^ ^43^ ^]^ Briefly, the reaction mixture containing 20 mM Tris‐Cl, pH 7.5, 150 mM NaCl, 10 mM MgCl_2_, 0.05% BSA, 2 mM β‐Mercaptoethanol, 4 ng IDH1 WT/K72ac 0.35 mM isocitrate were started by adding 0.25 µM NADP+. The IDH1‐produced NADPH was measured by the diaphorase/resazurin system, which converts resazurin into resorufin (excitation 550 nm, emission 585 nm) that was quantitatively analyzed by a plate reader (CLARIOstar Plus, BMG Labtech, German).

ALDH1A1 activity assays were performed according to the protocol that was previously reported.^[^ ^43^ ^]^ Briefly, the reaction mixture containing 50 mM Tris‐Cl, pH 7.5, 200 mM NaCl, 10 mM KCl, 2 mM DTT, 200 ng ALDH1A1 WT/K113ac/K193ac, 1 mM acetaldehyde were started by adding 250 µM NAD^+^. The ALDH1A1‐produced NADH was measured by the diaphorase/resazurin system, which converts resazurin into resorufin (excitation 550 nm, emission 585 nm) that was quantitatively analyzed by plate reader (CLARIOstar Plus, BMG Labtech, German). The concentrations of recombinant proteins were determined by Pierce™ Rapid Gold BCA Protein Assay Kit (Cat#A53225, Thermo Fisher Scientific, Rockford, USA).

### Crystallization, Data Collection, and Structure Determination

To obtain the co‐crystal of KARS70–580 bound to *N* ^6^‐acetyl‐ *l* ‐lysine (AcK), the protein solution of KARS_70–580_ was first preincubated with synthetic AcK (nKARS_70–580_: nAcK = 1:10) and 5 mM ATP for 1 h at 4 °C. The initial crystallization screening was carried out by the sitting‐drop vapor‐diffusion method at 16 °C using commercial screening kits. Crystals were observed after 7–8 days in a condition containing 0.06 M Morpheus Divalents, 0.1 m Morpheus Buffer system 1, pH 6.5, and 30% Morpheus Precipitant Mix 2. Single crystals were obtained by the seeding method. Crystals were harvested and subsequently soaked in mother liquor supplemented with 5 mM AcK and 5 mM ATP for ≈11 h. The crystals were cryoprotected with the reservoir solution supplemented with 20% (v/v) glycerol and then flash‐cooled in liquid nitrogen. The space group of the crystal was P2_1_2_1_2_1_, with cell dimensions of *a* = 130 Å, *b* = 90 Å, and *c* = 107 Å. There were two molecules in the asymmetric unit with a 53% solvent content. KARS_70–580_ apo form crystals were obtained in a similar condition.

X‐ray diffraction data were collected from a single crystal on beamlines BL17U1 and BL18U1 of the National Facility for Protein Science in Shanghai (NFPS) synchrotron Radiation Facility and processed with XDS and AIMLESS.^[^ ^44^ ^]^ The small molecule in the crystal of KARS complex was extracted by 80% methanol in purified water and identified by LC‐MS/MS spectra. The LC‐MS/MS method was the same used in the analysis of *N* ^6^‐(acetyl‐d_3_)‐ *l* ‐lysine in serum. The structure of KARS_70–580_‐AcK complex was determined and refined using the Phenix suite program.^[^ ^45^ ^]^ The structure of human KARS (PDB ID: 3BJU) was processed by the Sculptor program. Two ensembles, one containing residue 300–430 and the other comprising the rest part in chain A of 3BJU, were used for MR search. Model building was performed using Coot.^[^ ^46^ ^]^ Model refinement was carried out with Phenix‐Refine.^[^ ^47^ ^]^ Ramachandran plot and the quality of the structure were evaluated with MolProbity.^[^ ^48^ ^]^ The final models were deposited in the Protein Data Bank with accession codes 7VWQ (KARS‐apo) and 8XP4 (KARS‐AcK). PyMOL was used to present the structure. Refinement statistics were summarized in Table S6, Supporting Information.

### Thermofluor Shift Assay

SYPRO Orange Protein Gel Stain (Cat#S5692, Sigma‐Aldrich, St. Louis, USA) were diluted 1:2500 into the assay buffer (150 mM NaCl, 200 mM Tris‐Cl pH 7.5, 10 mM MgCl_2_, 20 mM β‐Mercaptoethanol). ALDH1A1 (WT/K113ac/K193ac) or IDH1 (WT/K72ac) was added with the final concentration of 0.2 µM. The 50 µL mixture was loaded into PCR 8‐Strip Tubes (Cat#CP0101, GSBIO, Wuxi, China). Melting curves were obtained by applying a temperature gradient from 25 to 99 °C and a heating rate of 0.5 °C/s. The fluorescence intensity was measured by a CFX Connect™ Real‐Time PCR System (Cat#1855201, Bio‐Rad, USA).

### MNase Assay

Chromatin decondensation was tested as previously described.^[^ ^16^ ^]^ Nuclei extractions were performed by using the Nuclei PURE Prep kit (Cat#NUC201, Sigma‐Aldrich, Shanghai, China). For MNase assay, 5 µL nuclei were digested with micrococcal nuclease (Cat#M0247S, NEB, MA, USA) at 37 °C for gradient time. The digestion was stopped by adding 0.5 M EDTA (pH 8.0). To analyze the digested genomic DNA, proteinase K(NEB) and RNase A (Solarbio) were added to the digestion products which were incubated at 37 °C for 1 h to remove protein and RNA. Genomic DNA were purified using phenol–chloroform extraction and then analyzed with 1.5% agarose gel.

### Analysis of the surface accessibility of coTM‐acetylation exclusive sites

By using the protein ID in the coTM‐exclusive acetylome, structure files of the candidate proteins were retrieved from the Protein Data Bank (https://www.rcsb.org/). The spatial position of the candidate lysine sites in the candidate proteins were analyzed by using Swiss‐PdbViewer 4.1.0 software. Lysine residues with accessibility to the surface <30% were termed as buried inside, otherwise termed as outside. Proteins without available structure in PDB were alternatively analyzed via their homolog protein structures and alphafold‐predicted structures (https://alphafold.ebi.ac.uk/).

### Immunoblotting

Proteins were extracted from cultured cells using 0.5% SDS lysis buffer containing protease, deacetylase, and phosphatase inhibitor cocktail. Immunoblotting analyses with indicated antibodies were performed as described previously.^[^ ^49^ ^]^

### Cell‐Free Protein Expression Coupled with *N* ^6^‐acetyl‐ *l* ‐Lysine

The cell‐free protein expression assays were performed by using the TnT Quick Coupled Transcription/Translation System (Cat#L1171, Promega, Madison, USA), according to the manufacturer's guideline with minor modifications. Briefly, the DNA template for luciferase expression was used as the positive control of DNA template. Gradient titrations of *N* ^6^‐acetyl‐*L*‐lysine were added to the assays. Protein synthesis reaction was carried out at 30 °C for 2 h. The protein expression level of luciferase was detected by immunoblotting assay with the antibody against luciferase (rabbit, Cat#A18259, lot# 3501634001, Abclonal, Wuhan, China). The acetylation level of the synthesized luciferase was measured by immunoblotting assay with the antibody against pan‐acetylation lysine.

To study the incorporation of d_3_‐AcK into the synthesized protein, 100 mM d_3_‐AcK was added to the cell‐free protein expression assay. The products of the in vitro protein expression reactions were collected and trypsin‐digested for MS/MS analysis.

### Analysis of L‐lysyl‐tRNA and *N* ^6^‐acetyl‐ *l* ‐lysyl‐tRNA by Northern Blot Hybridization

KARS‐catalyzed reactions were performed by mixing 10 mM Lysine or *N* ^6^‐acetyl‐ *l* ‐lysine, 1 mM ATP, 3 mg mL^−1^ tRNA, and 40 µg KARS that were then incubated at 37 °C for 2 h. The produced L‐lysyl‐tRNA and *N* ^6^‐acetyl‐ *l* ‐lysyl‐tRNA were separated from total tRNAs with acid‐urea polyacrylamide gel electrophoresis and detected as previously described.^[^ ^50^ ^]^ Briefly, 4 µg of total tRNA mix sample was loaded to a 12% gel for electrophoresis with a constant voltage of 100 V at 4 °C for 8.5 h. The tRNA was transferred onto a nylon membrane by TRANS‐Blot SD (Cat#170‐3940, Bio‐Rad, USA) at 650 mA for 30 min and the membrane was washed briefly in 5 × SSC (0.75 m sodium chloride, 0.075 m sodium citrate, adjusted to pH 7.0 with 1 N HCl) and cross‐linked by UV using 120 mJ. The membrane was hybridized overnight with Cy5.5 labeled tRNA probe at 30 °C. The signal was scanned and collected by an Odyssey CLX for fluorescence detection after the membrane was washed by washing buffer (6 × SSC, 0.1% SDS) twice.

### Pathway Enrichment

Kyoto Encyclopedia of Genes and Genomes (KEGG) pathway enrichment analysis was performed via DAVID (https://david.ncifcrf.gov/) and visualized using the Enrichment Map v3.3.3^[^ ^51^ ^]^ and Files Layout Algorithms v1.1.1 plugin of Cytoscape v3.8.2.^[^ ^52^ ^]^

### Statistical Analysis

Unless otherwise specified, data were processed using GraphPad Prism 9 for statistical analysis. All biological assay data were presented as the mean ± SD. The significance of differences in the experimental data was determined using Student's t‐test (two‐tailed) unless specifically indicated. Differences in means were considered statistically significant at *p <* 0.05 or *p <* 0.01.

## Conflict of Interest

The authors declare that they have no conflict of interest.

## Author Contributions

D.G., N.L., and X.Z. contributed equally to this work. Y.W. conceived and designed the study and wrote the manuscript. D.G., X.Z., W.Y., Y.W., F.T., S.Y., and S.L. performed biochemical experiments to identify the KARS activities in vivo and in vitro. J.H., X.P.D., C.H., and C.H., performed HPLC‐MS/MS to quantitatively analyze *N* ^6^‐acetyl‐ *l* ‐lysine and L‐lysine in cells and blood samples. R.Z. and K.L. performed bioinformatics analyses. X.X. and L.W. performed the chemical synthesis of *N* ^6^‐(acetyl‐d_3_)‐ *l* ‐lysine. M.F. and S.F. performed proteomic analyses. X.Z., N.L., Y.R.G., and Z.O. performed structural analyses.

## Supporting information



Supporting Information

Supporting Information

Supporting Information

Supporting Information

Supporting Information

Supporting Information

Supporting Information

Supporting Information

## Data Availability

The data that support the findings of this study are available in the supplementary material of this article.

## References

[1] X. Li , D. Zhang , C. Ren , Y. Bai , M. Ijaz , C. Hou , L. Chen , Compr. Rev. Food Sci. F 2021, 20, 289. 10.1111/1541-4337.12668 33443799

[2] C. L. Smith‐Hammond , K. N. Swatek , M. L. Johnston , J. J. Thelen , J. A. Miernyk , J. Proteomics 2014, 96, 56. 24211405 10.1016/j.jprot.2013.10.038

[3] S. Jiang , Y. Liu , Z. Shen , B. Zhou , Q. W. Shen , J. Proteomics 2019, 205, 103412. 31176012 10.1016/j.jprot.2019.103412

[4] J. Miedzianka , A. Zambrowicz , M. Zielinska‐Dawidziak , W. Drozdz , A. Nems , Molecules 2021, 26, 1575. 33809328 10.3390/molecules26061575 PMC8002035

[5] J. Miedzianka , A. Peksa , M. Aniolowska , Food Chem. 2012, 133, 1283.

[6] A. K. Jaiswal , Food processing technologies: impact on product attributes, CRC Press, USA 2017.

[7] M. C. Fan , et al., Food Hydrocoll. 2019, 96, 604.

[8] C. B. Zhao , H. Zhang , X. Y. Xu , Y. Cao , M. Z. Zheng , J. S. Liu , F. Wu , Process Biochem. 2017, 57, 117.

[9] M. Shvedunova , A. Akhtar , Nat Rev Mol Cell Bio 2022, 23, 329. 35042977 10.1038/s41580-021-00441-y

[10] J. Zierer , M. A. Jackson , G. Kastenmüller , M. Mangino , T. Long , A. Telenti , R. P. Mohney , K. S. Small , J. T. Bell , C. J. Steves , A. M. Valdes , T. D. Spector , C. Menni , Nat. Genet. 2018, 50, 790. 29808030 10.1038/s41588-018-0135-7 PMC6104805

[11] R. Gonzalez‐Dominguez , O. Jauregui , M. I. Queipo‐Ortuno , C. Andres‐Lacueva , Anal. Chem. 2020, 92, 13767. 32966057 10.1021/acs.analchem.0c02008

[12] R. Dallmann , A. U. Viola , L. Tarokh , C. Cajochen , S. A. Brown , P Natl Acad Sci. USA 2012, 109, 2625. 10.1073/pnas.1114410109 PMC3289302 22308371

[13] E. Verdin , M. Ott , Nat. Rev. Mol. Cell Biol. 2015, 16, 258. 25549891 10.1038/nrm3931

[14] W. K. Paik , L. Bloch‐Frankenthal , S. M. Birnbaum , M. Winitz , J. Greenstein , Arch. Biochem. Biophys. 1957, 69, 56. 13445179 10.1016/0003-9861(57)90472-1

[15] M. Mendoza , G. Egervari , S. Sidoli , G. Donahue , D. C. Alexander , P. Sen , B. A. Garcia , S. L. Berger , Sci. Adv. 2022, 8, abj5688. 10.1126/sciadv.abj5688 PMC8782443 35061542

[16] J. J. Zhang , T. T. Fan , Y‐Z. Mao , J‐L. Hou , M. Wang , M. Zhang , Y. Lin , L. Zhang , G. Q. Yan , Y. P. An , J. Yao , C. Zhang , P. C. Lin , Y.‐Y Yuan , J. Y. Zhao , W. Xu , S. M. Zhao , Nat Metab 2021, 3, 859. 34140692 10.1038/s42255-021-00405-8

[17] H. Neumann , S. Y. Peak‐Chew , J. W. Chin , Nat. Chem. Biol. 2008, 4, 232 . 18278036 10.1038/nchembio.73

[18] X‐D. He , W. Gong , J. N. Zhang , J. Nie , C. F. Yao , F.‐S Guo , Y. Lin , X. H. Wu , F. Li , J. Li , W. C. Sun , E.‐D Wang , Y. P. An , H‐R. Tang , G. Q. Yan , P. Y. Yang , Y. Wei , Y‐Z. Mao , P. C. Lin , J. Y. Zhao , Y. Xu , W. Xu , S. M. Zhao , Cell Metab. 2018, 27, 151. 29198988 10.1016/j.cmet.2017.10.015

[19] T. Yanagisawa , T. Sumida , R. Ishii , C. Takemoto , S. Yokoyama , Nat. Struct. Mol. Biol. 2010, 17, 1136. 20729861 10.1038/nsmb.1889

[20] H. P. Harding , Y. Zhang , H. Zeng , I. Novoa , P. D. Lu , M. Calfon , N. Sadri , C. Yun , B. Popko , R. Paules , D. F. Stojdl , J. C. Bell , T. Hettmann , J. M. Leiden , D. Ron , Mol. Cell 2003, 11, 619. 12667446 10.1016/s1097-2765(03)00105-9

[21] W. V. Everson , K. E. Flaim , D. M. Susco , S. R. Kimball , L. S. Jefferson , Am J Physiol‐Cell Ph 1989, 256, C18. 10.1152/ajpcell.1989.256.1.C18 2492151

[22] M. Guo , M. Ignatov , K. Musier‐Forsyth , P. Schimmel , X. L. Yang , P Natl Acad Sci. USA 2008, 105, 2331. 10.1073/pnas.0712072105 PMC2268136 18272479

[23] Y. Guo , D. F. Li , H. N. Ji , J. T. Zheng , N. Y. Zhou , Appl Environ Microb. 2021, 87, 01965.

[24] T. Terada , O. Nureki , R. Ishitani , A. Ambrogelly , M. Ibba , D. Söll , S. Yokoyama , Nat Struct Biol 2002, 9, 257. 11887185 10.1038/nsb777

[25] T. Narita , B. T. Weinert , C. Choudhary , Nat Rev Mol Cell Bio 2019, 20, 156. 30467427 10.1038/s41580-018-0081-3

[26] P. Walter , D. Ron , Science 2011, 334, 1081. 22116877 10.1126/science.1209038

[27] A. Drazic , L. M. Myklebust , R. Ree , T. Arnesen , Bba‐Proteins Proteom. 2016, 1864, 1372. 10.1016/j.bbapap.2016.06.007 27296530

[28] N. A. Wolfson , C. A. Pitcairn , E. D. Sullivan , C. G. Joseph , C. A. Fierke , Anal. Biochem. 2014, 456, 61. 24674948 10.1016/j.ab.2014.03.012 PMC4470474

[29] T. Fujino , J. Kondo , M. Ishikawa , K. Morikawa , T. T. Yamamoto , J. Biol. Chem. 2001, 276, 11420. 11150295 10.1074/jbc.M008782200

[30] A. Luong , V. C. Hannah , M. S. Brown , J. L. Goldstein , J. Biol. Chem. 2000, 275, 26458. 10843999 10.1074/jbc.M004160200

[31] G. Figurelia , P. Willnow , A. A. Teleman , Dev. Cell 2020, 54, 156. 32693055 10.1016/j.devcel.2020.06.036

[32] A. E. Boukouris , S. D. Zervopoulos , E. D. Michelakis , Trends Biochem. Sci. 2016, 41, 712. 27345518 10.1016/j.tibs.2016.05.013

[33] S. Sivanand , I. Viney , K. E. Wellen , Trends Biochem. Sci. 2018, 43, 61. 29174173 10.1016/j.tibs.2017.11.004 PMC5741483

[34] V. Bulusu , S. Tumanov , E. Michalopoulou , N. J. van den Broek , G. MacKay , C. Nixon , S. Dhayade , Z. T. Schug , J. Vande Voorde , K. Blyth , E. Gottlieb , A. Vazquez , J. J. Kamphorst , Cell Rep. 2017, 18, 647. 28099844 10.1016/j.celrep.2016.12.055 PMC5276806

[35] C. Q. Ye , B. P. Tu , Trends Endocrin Met. 2018, 29, 626. 10.1016/j.tem.2018.06.002 PMC6109460 30001904

[36] M. Shvedunova , A. Akhtar , Nat. Rev. Mol. Cell Biol. 2022, 23, 329. 35042977 10.1038/s41580-021-00441-y

[37] B. R. Sabari , D. Zhang , C. D. Allis , Y. M. Zhao , Nat Rev Mol Cell Bio 2017, 18, 90. 27924077 10.1038/nrm.2016.140 PMC5320945

[38] T. C. S. Ho , A. H. Y. Chan , A. Ganesan , J. Med. Chem. 2020, 63, 12460. 32608981 10.1021/acs.jmedchem.0c00830

[39] J. Baeza , J. A. Dowell , M. J. Smallegan , J. Fan , D. Amador‐Noguez , Z. Khan , J. M. Denu , J. Biol. Chem. 2014, 289, 21326. 24917678 10.1074/jbc.M114.581843 PMC4118097

[40] M. A. Mune Mune , S. R. Minka , I. L. Mbome , Int. J. Food Sci. Nutrit. 2011, 62, 310. 21271839 10.3109/09637486.2010.538670

[41] L. Li , X. Xu , K. Lv , G. Zheng , H. Wang , S. Chen , L. Huang , Y. Liu , Y. Zhang , Z. Tang , L. Zhang , J. Wang , J. Qiao , H. Li , X. Wang , G. Yao , C. Fang , Brit J. Pharmacol. 2023, 180, 287. 36166754 10.1111/bph.15964

[42] M. Itoh , H. Dai , S. I. Horike , J. Gonzalez , Y. Kitami , M. Meguro‐Horike , I. Kuki , S. Shimakawa , H. Yoshinaga , Y. Ota , T. Okazaki , Y. Maegaki , S. Nabatame , S. Okazaki , H. Kawawaki , N. Ueno , Y.‐I Goto , Y. Kato , Brain: J. Neurol. 2019, 142, 560. 10.1093/brain/awz001 30715177

[43] F. Wang , J. Travins , B. DeLaBarre , V. Penard‐Lacronique , S. Schalm , E. Hansen , K. Straley , A. Kernytsky , W. Liu , C. Gliser , H. Yang , S. Gross , E. Artin , V. Saada , E. Mylonas , C. Quivoron , J. Popovici‐Muller , J. O. Saunders , F. G. Salituro , S. Yan , S. Murray , W. Wei , Y. Gao , L. Dang , M. Dorsch , S. Agresta , D. P. Schenkein , S. A. Biller , S. M. Su , S. de Botton , et al., Science 2013, 340, 622. 23558173 10.1126/science.1234769

[44] W. Kabsch , Acta Crystallogr. D Biol. Crystallogr. 2010, 66, 133. 20124693 10.1107/S0907444909047374 PMC2815666

[45] P. D. Adams , P. V. Afonine , G. Bunkóczi , V. B. Chen , I. W. Davis , N. Echols , J. J. Headd , L.‐W Hung , G. J. Kapral , R. W. Grosse‐Kunstleve , A. J. McCoy , N. W. Moriarty , R. Oeffner , R. J. Read , D. C. Richardson , J. S. Richardson , T. C. Terwilliger , P. H. Zwart , Acta Crystallogr D 2010, 66, 213. 20124702 10.1107/S0907444909052925 PMC2815670

[46] P. Emsley , B. Lohkamp , W. G. Scott , K. Cowtan , Acta Crystallogr. D Biol. Crystallogr. 2010, 66, 486. 20383002 10.1107/S0907444910007493 PMC2852313

[47] P. V. Afonine , R. W. Grosse‐Kunstleve , N. Echols , J. J. Headd , N. W. Moriarty , M. Mustyakimov , T. C. Terwilliger , A. Urzhumtsev , P. H. Zwart , P. D. Adams , Acta Crystallogr D 2012, 68, 352. 22505256 10.1107/S0907444912001308 PMC3322595

[48] V. B. Chen , W. B. Arendall 3rd , J. J. Headd , D. A. Keedy , R. M. Immormino , G. J. Kapral , L. W. Murray , J. S. Richardson , D. C. Richardson , Acta Crystallogr. D Biol. Crystallogr. 2010, 66, 12. 20057044 10.1107/S0907444909042073 PMC2803126

[49] Y. Wang , Y. R. Guo , K. Liu , Z. Yin , R. Liu , Y. Xia , L. Tan , P. Yang , J‐H. Lee , X. J. Li , D. Hawke , Y. Zheng , X. Qian , J. Lyu , J. He , D. Xing , Y. J. Tao , Z. Lu , Nature 2017, 552, 273. 29211711 10.1038/nature25003 PMC5841452

[50] B. D. Janssen , E. J. Diner , C. S. Hayes , Methods Molecul, Biol. 2012, 905, 291. 10.1007/978-1-61779-949-5_19 PMC3682404 22736012

[51] D. Merico , R. Isserlin , O. Stueker , A. Emili , G. D. Bader , PLoS One 2010, 5, 13984. 10.1371/journal.pone.0013984 PMC2981572 21085593

[52] P. Shannon , A. Markiel , O. Ozier , N. S. Baliga , J. T. Wang , D. Ramage , N. Amin , B. Schwikowski , T. Ideker , Genome Res. 2003, 13, 2498. 14597658 10.1101/gr.1239303 PMC403769

