# Supplementary material for: Co‐Translational Deposition of N 6‐Acetyl‐L‐Lysine in Nascent Proteins Contributes to the Acetylome in Mammalian Cells
Source: Adv Sci (Weinh). 2024 Dec 4;12(4):2403309. doi: 10.1002/advs.202403309 (PMC11789599; doi:10.1002/advs.202403309)
Supplement: Supplementary file 1 — Supporting Information [file ADVS-12-2403309-s008.pdf]

ADVANCED SCIENCE
Open Access

Supporting Information

for *Adv. Sci.*, DOI 10.1002/advs.202403309

Co-Translational Deposition of N^6-Acetyl-*L*-Lysine in Nascent Proteins Contributes to the Acetylome in Mammalian Cells

Dingyuan Guo, Nan Li, Xiaoyan Zhang, Runxin Zhou, Jie He, Xiao-Ping Ding, Weixing Yu, Fuqiang Tong, Sibi Yin, Yu Wang, Xin Xu, Long Wang, Mingzhu Fan, Shan Feng, Ke Liu, Ke Tang, Zhuqing Ouyang, Yusong R Guo and Yugang Wang**

Supplementary Figures

Fig S1

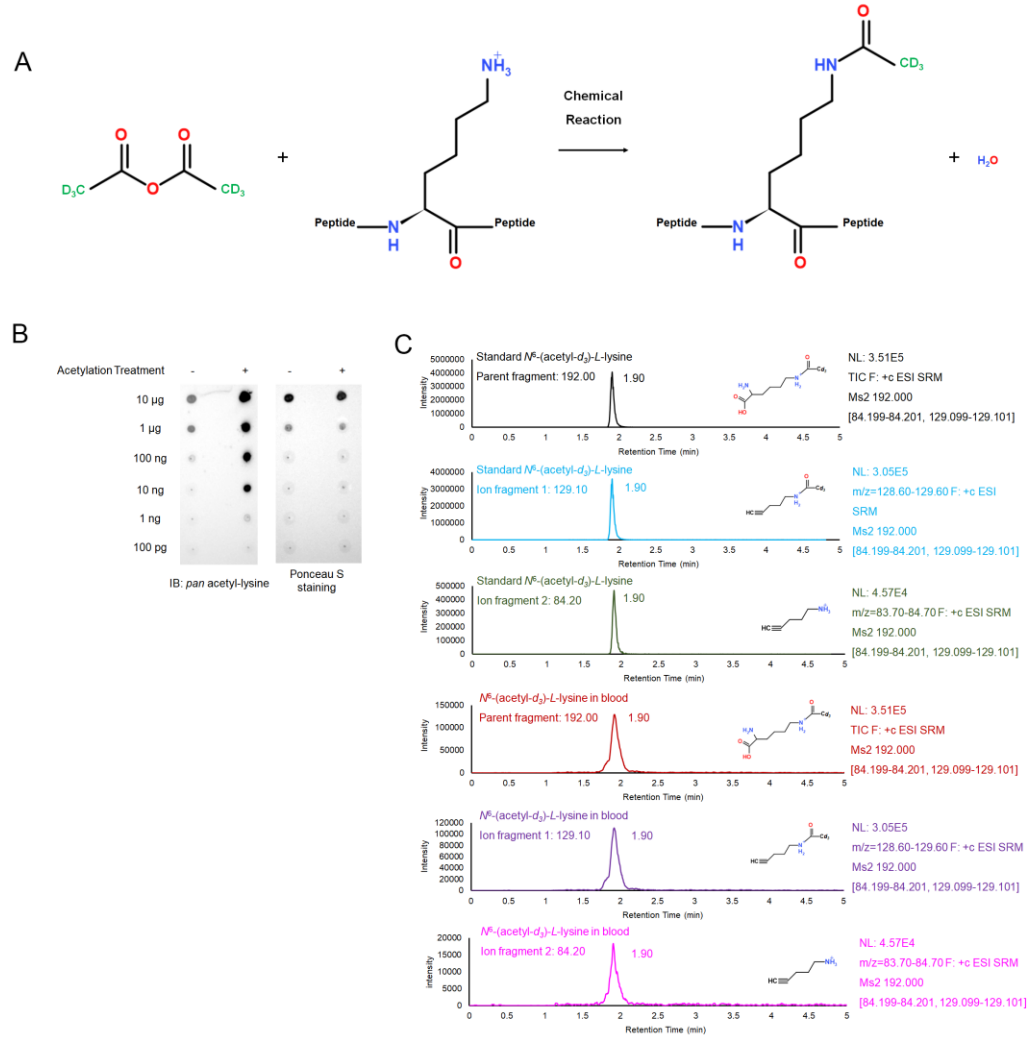

Figure S1. N^6-acetyl-*L*-lysine residues in diet contributes the acetylome of diet-consumer.

(**A**), Illustration of the workflow of synthesizing deuterium-labelled acetylated dietary protein (N^6-(acetyl-d_3)-*L*-lysine-protein).

(**B**), Validation of the synthesized deuterium-labelled acetylated dietary protein by performing immunoblotting assay with the antibody against acetyl-lysine. Representative images of triplicate experiments are shown.

(**C**), Identification of d_3-AcK in the blood from mice fed with N^6-(acetyl-d_3)-*L*-lysine-protein. The parent ion and other two ion fragments of synthesized d_3-AcK have similar elution profiles with that of d_3-AcK identified in the blood from mice fed with N^6-(acetyl-d_3)-*L*-lysine-protein. The y-axis indicates the signal intensity, the x-axis indicates the retention time (minutes).

Fig S2

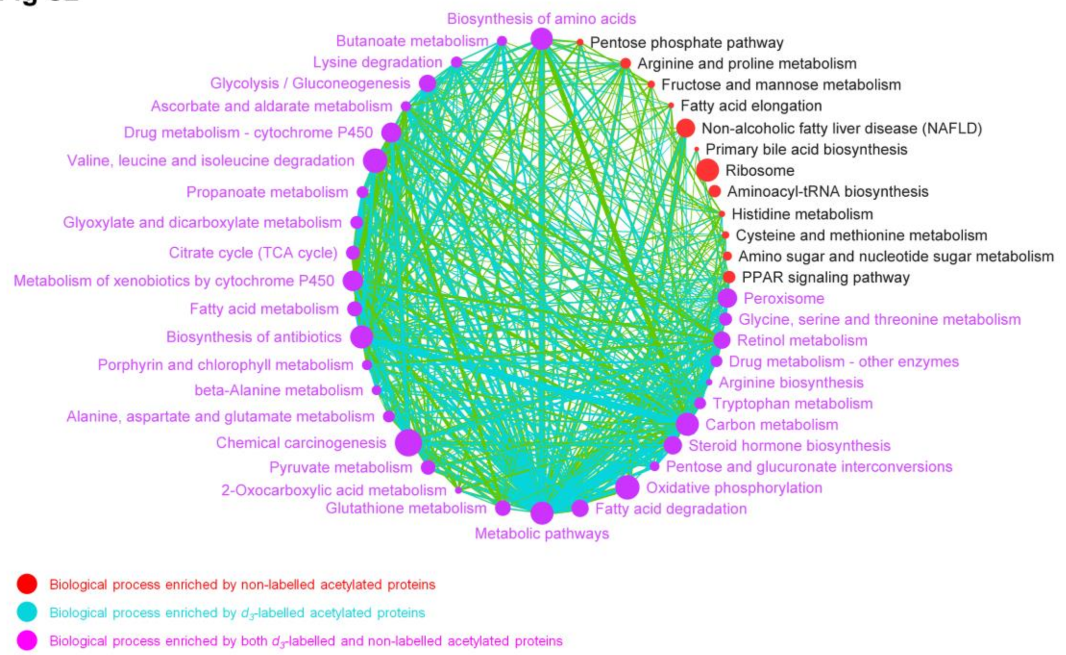

Figure S2 Biological processes enriched by proteins with deuterium-labelled acetylation in mice liver.

Nodes stand for KEGG defined pathways (P<0.001 and FDR<0.001). Each node represents one biological process. The node size reflects the number of proteins in the pathway. Two nodes are connected if there were one or more proteins included in both nodes. Biological processes that are enriched by unlabeled acetylation proteins (red), deuterium-labelled acetylation proteins (cyan), and proteins in both coTM-acetylome and PTM-acetylomes (plum) are highlighted in colors.

Fig S3

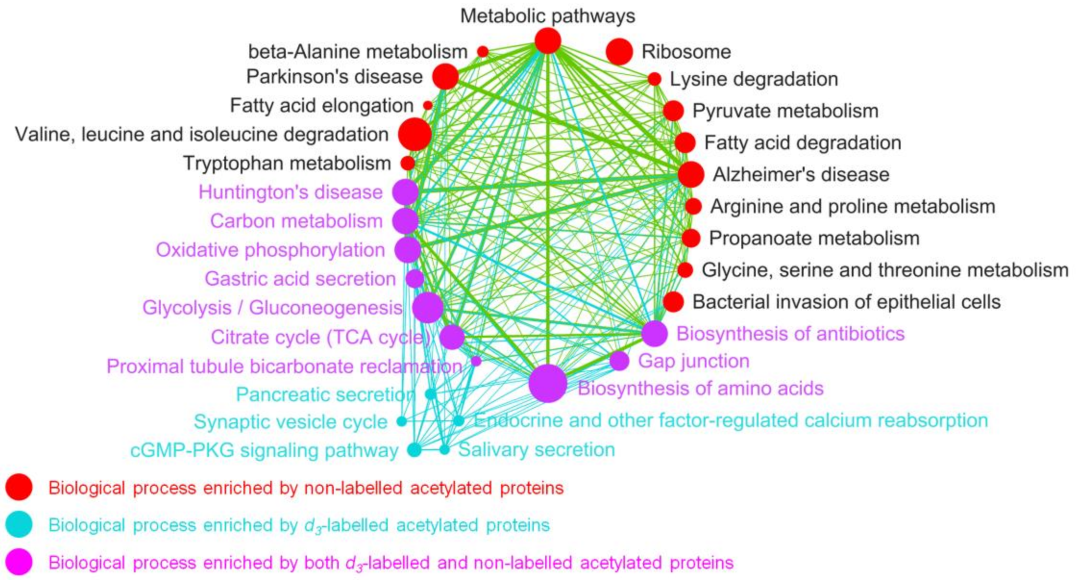

Figure S3. Biological processes enriched by proteins with deuterium-labelled acetylation in mice brain.

Nodes stand for KEGG defined pathways (P<0.001 and FDR<0.001). Each node represents one biological process. The node size reflects the number of proteins in the pathway. Two nodes are connected if there were one or more proteins included in both nodes. Biological processes that are enriched by unlabeled acetylation proteins (red), deuterium-labelled acetylation proteins (cyan), and proteins in both coTM-acetylome and PTM-acetylomes (plum) are highlighted in colors.

Fig S4

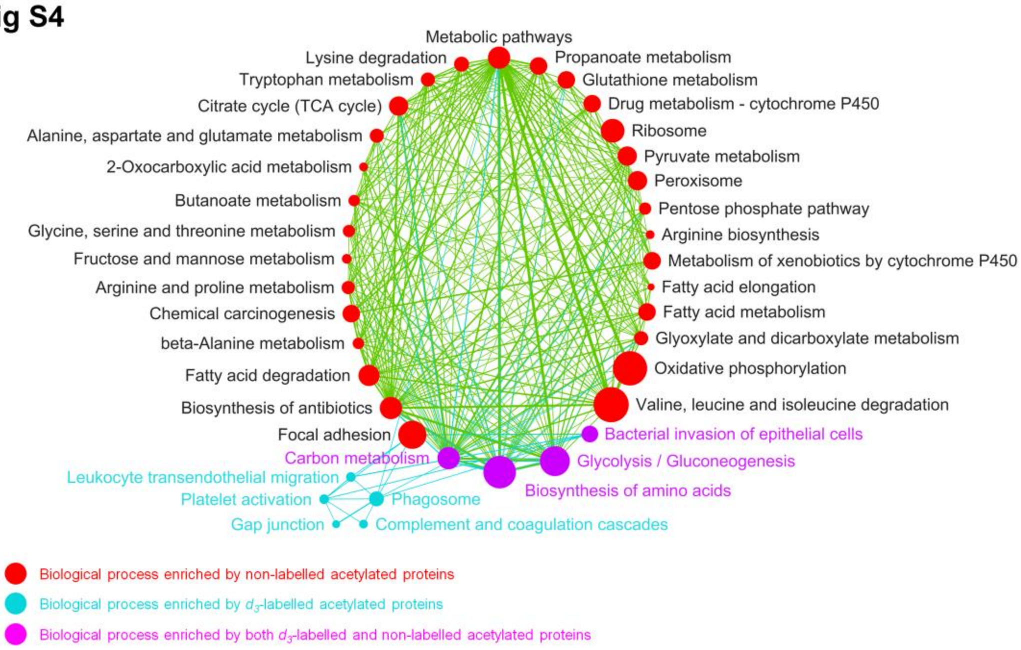

Figure S4. Biological processes enriched by proteins with deuterium-labelled acetylation in mice lung.

Nodes stand for KEGG defined pathways ($P<0.001$ and FDR<0.001). Each node represents one biological process. The node size reflects the number of proteins in the pathway. Two nodes are connected if there were one or more proteins included in both nodes. Biological processes that are enriched by unlabeled acetylation proteins (red), deuterium-labelled acetylation proteins (cyan), and proteins in both coTM-acetylome and PTM-acetylomes (plum) are highlighted in colors.

Fig S5

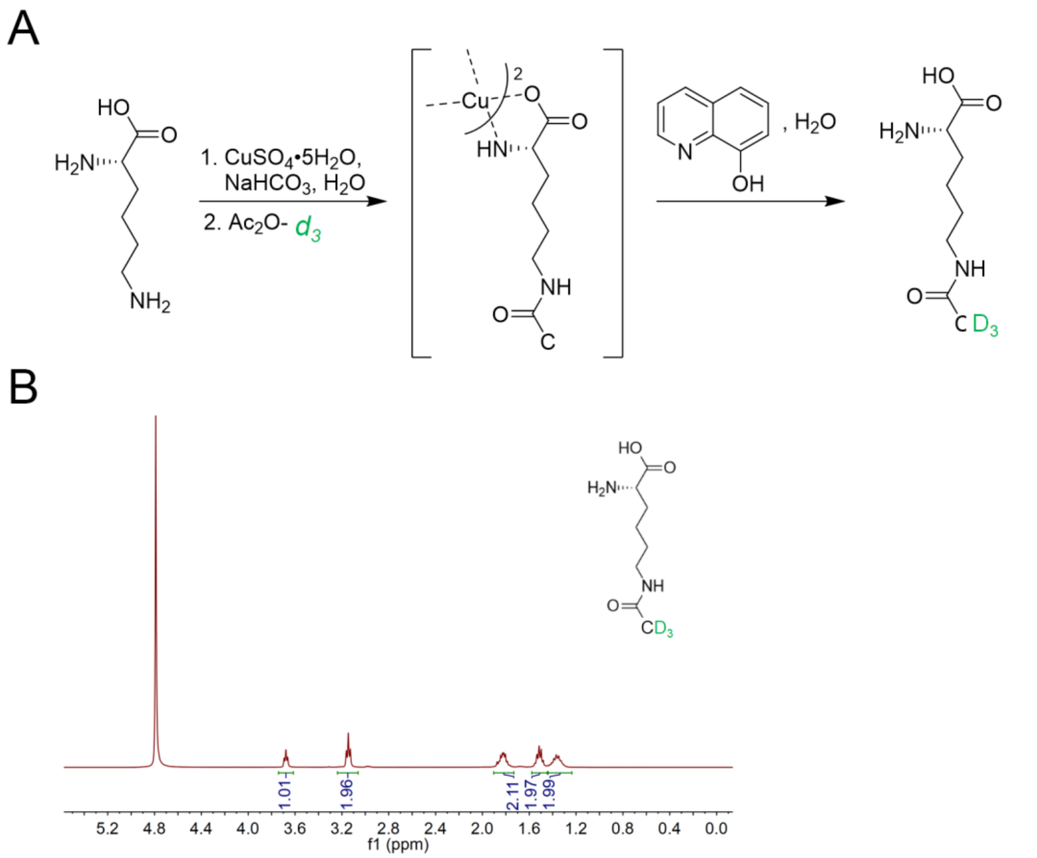

Figure S5. Synthesis of *N*⁶-(acetyl-*d*₃)-*L*-lysine

(**A**), Illustration of the workflow of synthesizing deuterium-labelled N^6-acetyl-*L*-lysine (d_3-AcK).

(**B**), Validation of the synthesized d_3-AcK. ^{1}H NMR (D_2O, 500 MHz, 295 K, ppm) spectrum of the synthesized d_3-AcK, recorded on the Agilent spectrometer.

Fig S6

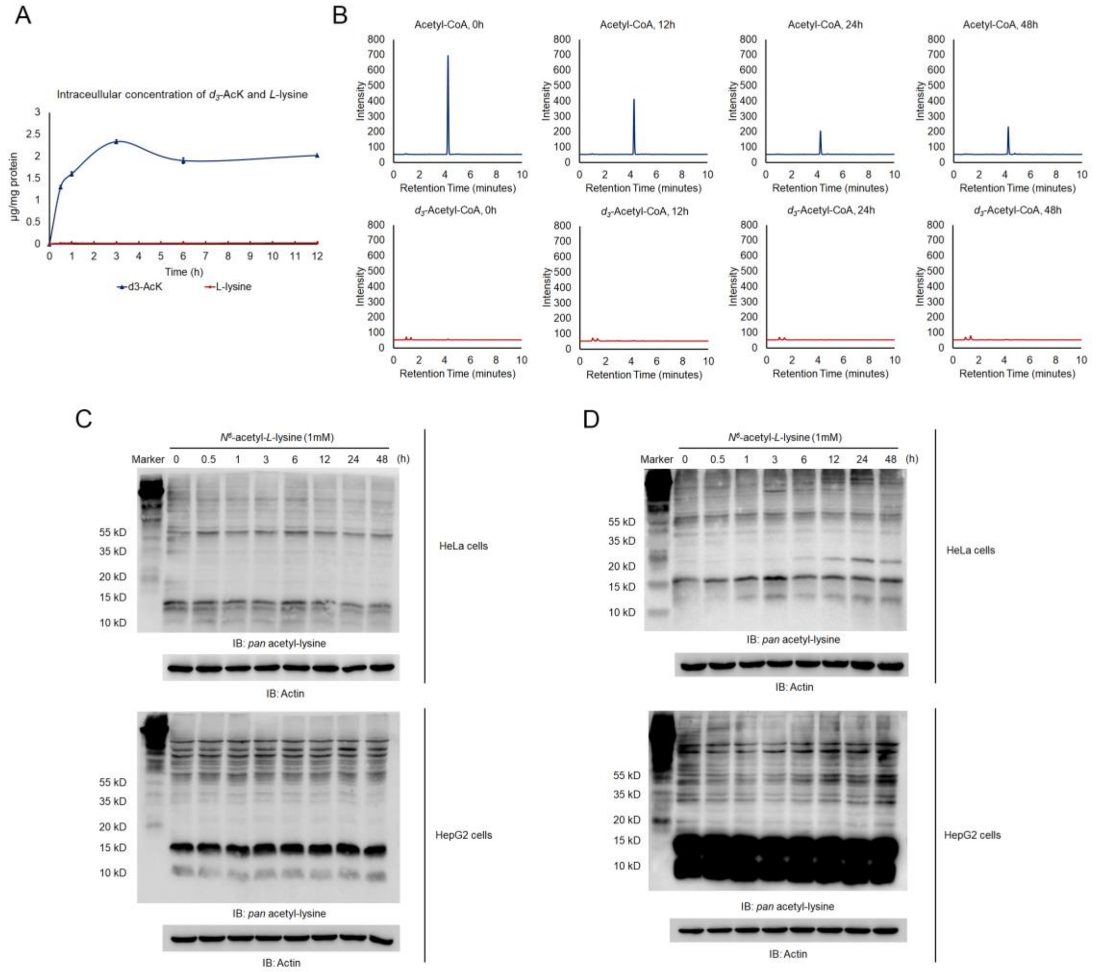

Figure S6. The effect of AcK on the acetylome of cells is harnessed by deacetylases

(**A**), Quantification of d_3-AcK and *L*-lysine in cells. Kinetic studies of d_3-AcK and *L*-lysine in $^{13}C_6$,$^{15}N_2$-*L*-lysine-labelled cells treated with d_3-AcK. Each data point is presented as the means±s.d. of three assays (n=3).

(**B**), Quantification of deuterium-labelled acetyl-CoA in cells. Kinetic studies of deuterium-labelled acetyl-CoA in cells treated with d_3-AcK. Representative chromatograph spectrum of triplicate experiments is shown. The y-axis indicates the signal intensity, the x-axis indicates the retention time (minutes).

(**C**), AcK treatment has no influence on the *pan*-acetylation of proteome in cells. The cultured HeLa and HepG2 cells were treated with 1 mM AcK for a series of time-points. The *pan*-acetylation of proteins in the treated cells were analyzed by immunoblotting assays with the indicated antibodies. Representative images of triplicate experiments are shown.

(**D**), AcK treatment increases the *pan*-acetylation of proteins in cells treated with deacetylase inhibitor cocktail. The cultured HeLa and HepG2 cells were treated with 1 mM AcK for a series of time-points. Deacetylase inhibitor cocktail is added in the experiments to block the surveillance of deacetylases that might possibly remove the AcK treatment-induced protein acetylation in cells. The *pan*-acetylation of proteins in the treated cells were analyzed by immunoblotting assays with the indicated antibodies. Representative images of triplicate experiments are shown.

Fig S7

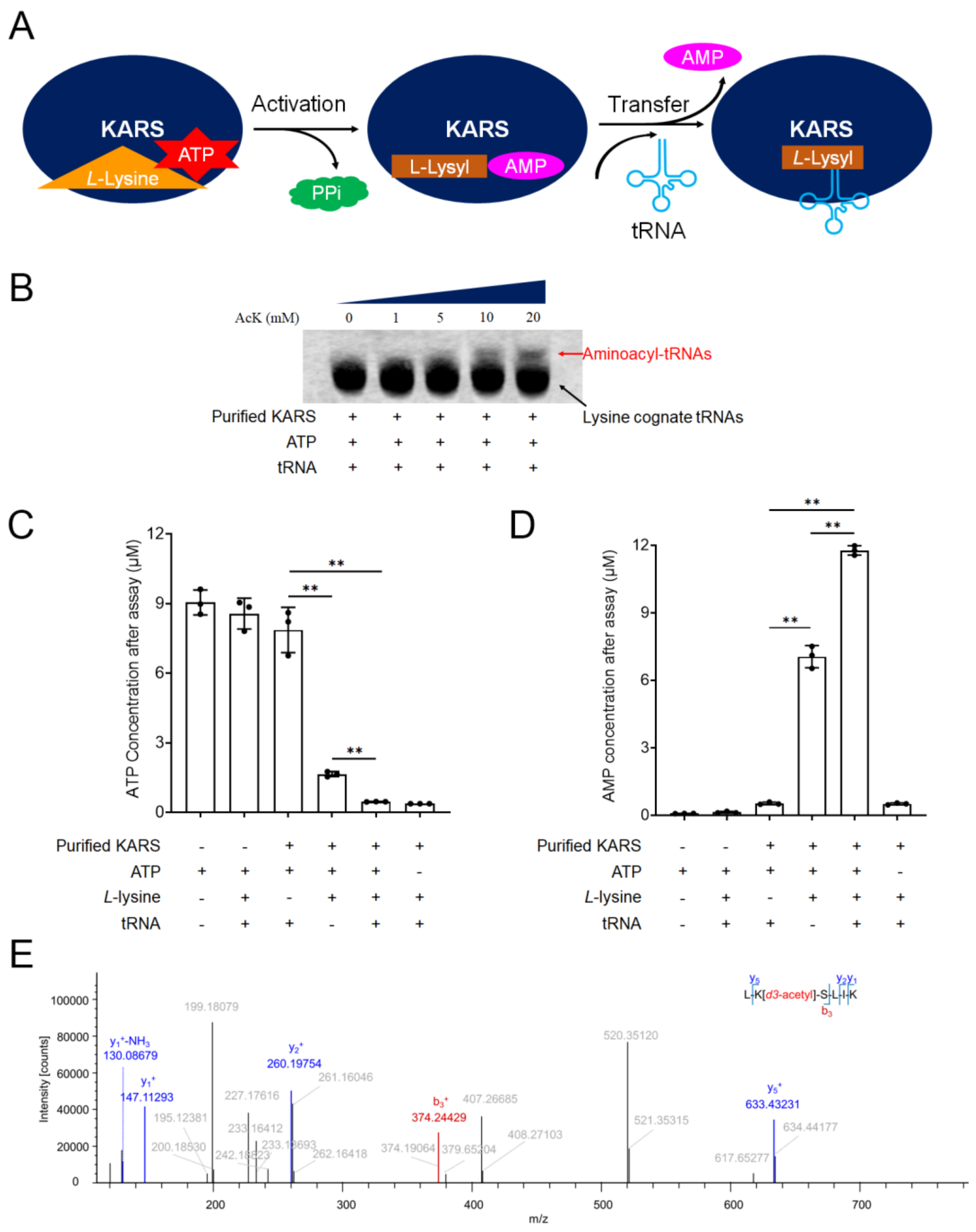

Figure S7. N^6-acetyl-*L*-lysine is an alternative substrate of KARS

(**A**), The scheme illustrating the two-step reaction catalyzed by KARS.

(**B**), AcK-dependent generation of aminoacyl-tRNAs catalyzed by KARS. Northern blotting analysis of lysine cognate tRNAs by using fluorescence-labelled single strand DNA probe that specifically recognizes lysine cognate tRNAs. The free lysine

cognate tRNA and aminoacyl-tRNAs are indicated with black and red arrows, respectively. Representative images of triplicate experiments are shown.

(**C**) and (**D**), KARS catalyzes ATP consumption and AMP production in the absence of tRNAs. Purified KARS was incubated with ATP and *L*-lysine for 1 hour. The ATP consumption (**C**) and AMP production (**D**) in the assays were measured and quantitatively analyzed. Two-sided *t*-test analyses were conducted. The data are presented as the means±s.d. of three independent experiments (n=3). $^{**}P<0.01$.

(**E**), Identification of d_3-AcK incorporated protein synthesized by cell-free protein translation system. d_3-AcK was added to the cell-free protein translation reaction that produces DNA-templated luciferase. MS/MS spectra of d_3-labelled acetylated peptide from the synthesized luciferase. The b ion refers to the N-terminal parts of the peptide, and the y ion refers to the C-terminal parts of the peptide. Data represent three experiments.

Fig S8

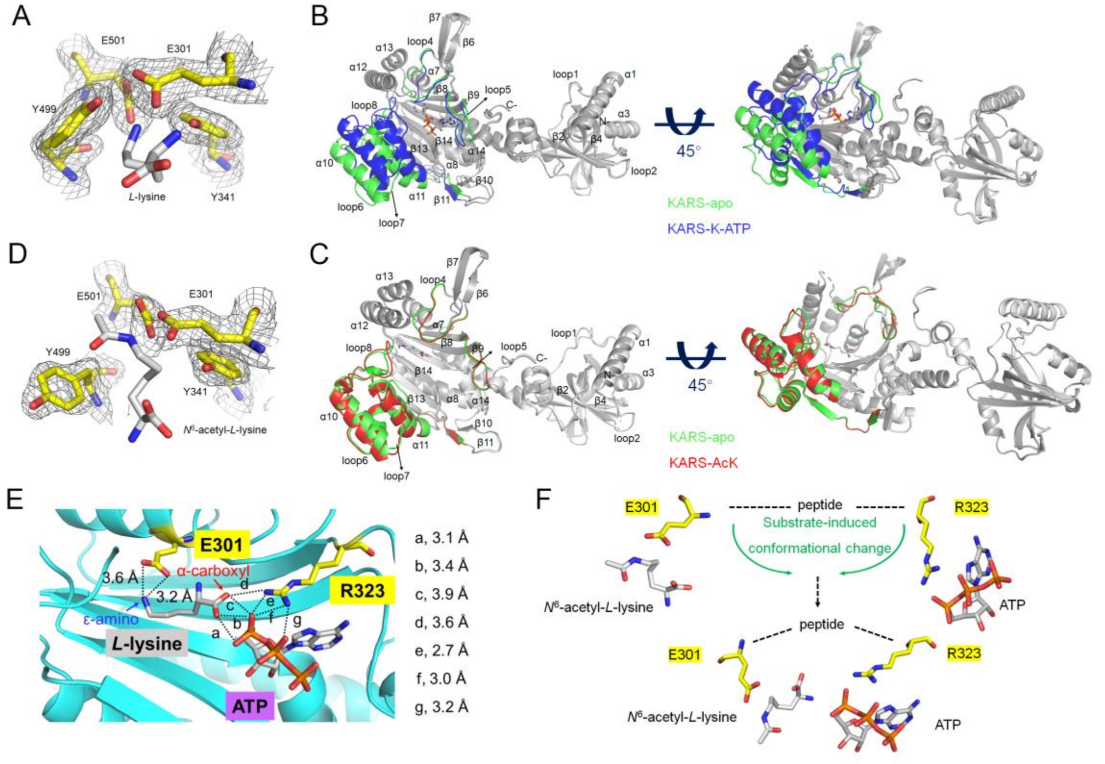

Figure S8. The molecular basis of KARS utilizing *N*^{6}-acetyl-*L*-lysine as substrate.

(**A**), The densities of the four clamping residues around *L*-lysine. The map is contoured at 1σ. Glu301, Tyr341, Tyr499 and Glu501 are shown as yellow sticks. The *L*-lysine is shown as gray sticks.

(**B**), Superimposition analysis of the structures of KARS-apo and KARS-K-ATP. Only the regions showing structural difference between the KARS-apo (green) and KARS-K-ATP (blue) are colored and labelled. *L*-Lysine and ATP in the structure of KARS-K-ATP are shown as sticks.

(**C**), Superimposition analysis of the structures of KARS-apo and KARS-AcK. Only the regions showing structural difference between the KARS-apo (green) and KARS-AcK (red) are colored and labelled. AcK in the structure of KARS-AcK (gray) are

shown as sticks.

(**D**), The densities of the four clamping residues around AcK. The map is contoured at 1σ. Glu301, Tyr341, Tyr499 and Glu501 are shown as yellow sticks. The AcK is shown as gray sticks.

(**E**), Analysis of the interactions between the substrates and the residues of KARS-E301 and KARS-R323. The substrates-surrounding regions of KARS-K-ATP structure is shown as cartoon in blue. *L*-lysine, ATP, and the KARS-E301-R323 residues are shown as sticks and labelled. The distance between the *L*-lysine, ATP and KARS-E301-R323 residues was measured and labelled.

(**F**), The scheme illustrating the roles of Arg-323 and Glu-301 in KARS-AcK reaction.

Fig S9

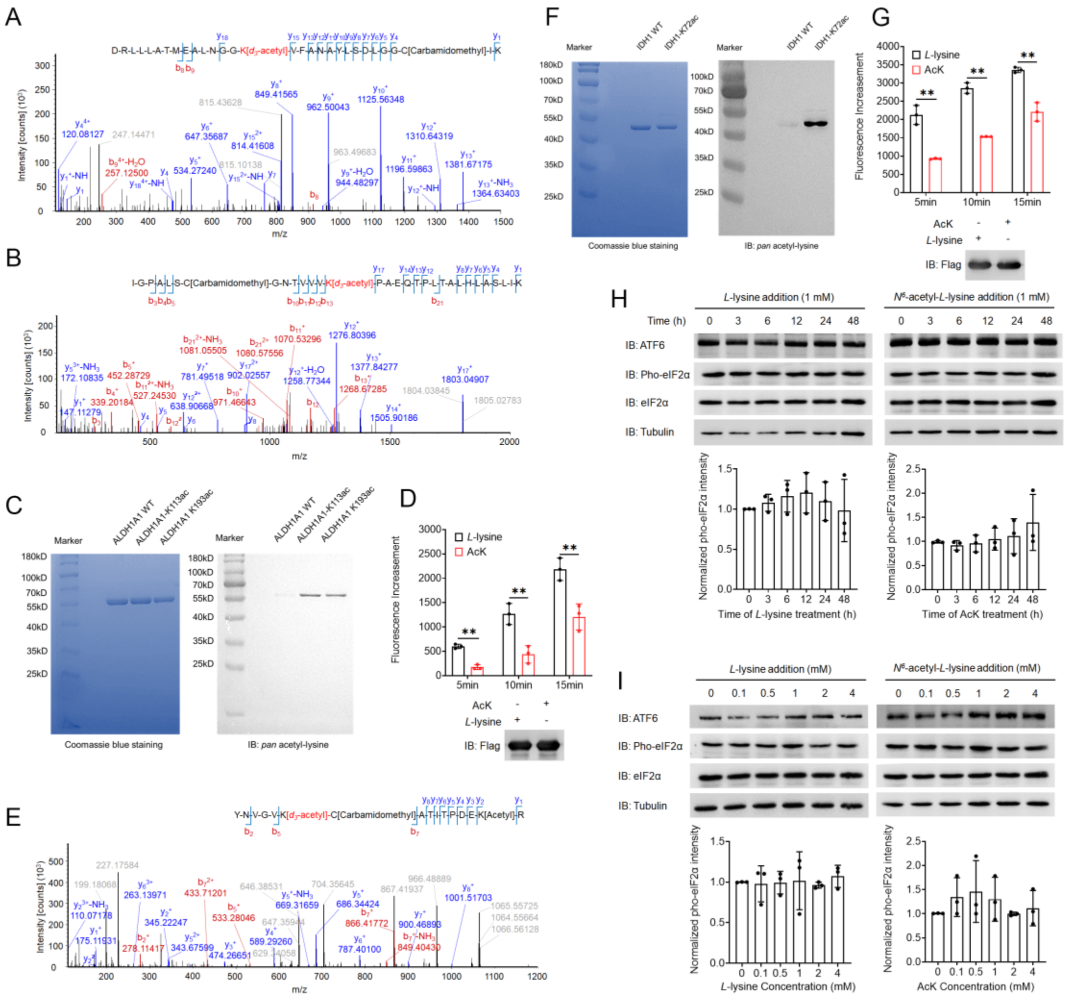

Figure S9. Deposition of N^6-acetyl-*L*-lysine in the buried regions of proteins

(**A**) and (**B**), Identification of deuterium-labelled acetylation sites that are buried inside ALDH1A1 protein. MS/MS spectra of ALDH1A1-K113 acetylation (**A**) and ALDH1A1-K193 acetylation (**B**). The b ion refers to the N-terminal parts of the peptide, and the y ion refers to the C-terminal parts of the peptide. Data represent two independent experiments.

(**C**), Expression and purification of recombinant ALDH1A1wild-type, ALDH1A1-K113ac, ALDH1A1-K193ac proteins. Coomassie blue staining (left) and immunoblotting assay (right) were performed to show the purity and acetylation of

studied proteins.

(**D**), The influence of AcK-treatment on the catalytic activity of ALDH1A1. Flag-ALDH1A1 was ectopically expressed and pull-downed from mammalian cells treated with *L*-lysine and AcK, respectively. The enzymatic activity assays were performed by measuring the NADH production in each assay. Each data is presented as the means±s.d. of three independent assays (n=3). Two-sided *t*-test analyses were conducted. $^{**}P<0.01$. The protein level of ALDH1A1 in each assay was tested by immunoblotting assay with indicated antibody. Representative images of triplicate experiments are shown.

(**E**), Identification of deuterium-labelled acetylation site that is buried inside IDH1 protein. MS/MS spectra of IDH1-K72 acetylation. The b ion refers to the N-terminal parts of the peptide, and the y ion refers to the C-terminal parts of the peptide. Data represent one experiment.

(**F**), Expression and purification of recombinant IDH1wild-type and IDH1-K72ac proteins. Coomassie blue staining (left) and immunoblotting assay (right) were performed to show the purity and acetylation of studied proteins.

(**G**), The influence of AcK-treatment on the catalytic activity of IDH1. The enzymatic activity assays of IDH1 pull-downed from mammalian cells treated with *L*-lysine and AcK were performed by measuring the NADPH production in each assay. Each data is presented as the means±s.d. of three independent assays (n=3). Two-sided *t*-test analyses were conducted. $^{**}P<0.01$. The protein level of IDH1 in each assay was

tested by immunoblotting assay with indicated antibody. Representative images of triplicate experiments are shown.

(**H**) and (**I**), The influence of AcK treatment on unfolded protein response (UPR). Cultured HeLa cells were treated with N^6-acetyl-*L*-lysine at a series of dosages for 48 hours (**H**) or at 1mM for a series of time-points (**I**). Immunoblotting assays were performed with the indicated antibodies. Representative images of triplicate experiments are shown. The statistical analysis of phosphorylated eIF2α of each set of samples is presented below the immunoblotting images. Each data is presented as the means ± s.d. of three independent assays (n=3). Two-sided *t*-test analyses were conducted. No statistical difference was found between the studied groups.
