# Supplementary material for: Co‐Translational Deposition of N 6‐Acetyl‐L‐Lysine in Nascent Proteins Contributes to the Acetylome in Mammalian Cells
Source: Adv Sci (Weinh). 2024 Dec 4;12(4):2403309. doi: 10.1002/advs.202403309 (PMC11789599; doi:10.1002/advs.202403309)
Supplement: Supplementary file 7 — Supporting Information [file ADVS-12-2403309-s004.pdf]

ADVANCED SCIENCE
Open Access

Supporting Information

for *Adv. Sci.*, DOI 10.1002/advs.202403309

Co-Translational Deposition of N^6-Acetyl-*L*-Lysine in Nascent Proteins Contributes to the Acetylome in Mammalian Cells

Dingyuan Guo, Nan Li, Xiaoyan Zhang, Runxin Zhou, Jie He, Xiao-Ping Ding, Weixing Yu, Fuqiang Tong, Sibi Yin, Yu Wang, Xin Xu, Long Wang, Mingzhu Fan, Shan Feng, Ke Liu, Ke Tang, Zhuqing Ouyang, Yusong R Guo and Yugang Wang**

Table S6. Crystallographic Statistics of KARS and KARS-AcK Complex Structures

	KARS(70-580)	KARS(70-580)-AcK
Wavelength (Å)	0.97915	0.97915
Resolution range (Å)	50.00 - 2.55 (2.59 - 2.55)	23.06 - 2.26 (2.341 - 2.26)
Space group	P 2_1 2_1 2_1	P 2_1 2_1 2_1
Unit cell (Å)	90.331 107.664 130.516 90 90 90	130.337 90.469 107.201 90 90 90
Total reflections	969761	798883
Unique reflections	42172 (4151)	114081 (5822)
Multiplicity	6.5(5.9)	13.4 (12.5)
Completeness (%)	99.70 (99.71)	99.58 (98.41)
Mean I/sigma(I)	13.5 (2.1)	7.77 (1.98)
Wilson B-factor (Å^2)	39.70	35.66
R-merge	0.152 (0.930)	0.2169 (1.224)
R-meas	0.165 (0.961)	0.2258 (1.277)
R-pim	0.064 (0.383)	0.06223 (0.3564)
CC1/2	0.991 (0.865)	0.994 (0.888)
Reflections used in refinement	42082 (4141)	59721 (5817)
Reflections used for R-free	1993 (197)	1989 (194)
R-work	0.2147 (0.2809)	0.2327 (0.3389)
R-free	0.2453 (0.3348)	0.2642 (0.2743)
Number of non-hydrogen atoms	8294	8193
Macromolecules	8111	7955
Ligands	0	26
solvent	183	212
Protein residues	999	966
RMS(bonds)	0.003	0.003
RMS(angles)	0.64	0.575
Ramachandran favored (%)	98.08	96.78
Ramachandran allowed (%)	1.92	3.22
Ramachandran outliers (%)	0.00	0.00
Rotamer outliers (%)	0.56	0.92
Clashscore	3.89	4.36
Average B-factor	51.37	53.0
Macromolecules	51.54	51.76
Ligand		62.21
solvent	43.78	44.50

Statistics for the highest-resolution shell are shown in parentheses.
